# Expert consensus on Lipoprotein(a) in the Gulf countries: Navigating cardiovascular risk and therapeutic advances

**DOI:** 10.1016/j.athplu.2026.100569

**Published:** 2026-05-07

**Authors:** Wael Almahmeed, Hani Sabbour, Ronney Shantouf, Asma Aljaberi, Hassan El Tamimi, Farhana Bin Lootah, Nasreen Al Sayed, Juwairia AlAli, Mousa Akbar, Abdullah Shehab, Ahmad AlSarraf, Khalid Al Waili, Thomas F. Lüscher

**Affiliations:** a Heart, Vascular and Thoracic Institute, Cleveland Clinic Abu Dhabi, Abu Dhabi, United Arab Emirates; b Department of Cardiology, Mediclinic Airport Road Hospital, Abu Dhabi, United Arab Emirates; c Department of Medicine, Division of Endocrinology, Tawam Hospital, Al Ain, Abu Dhabi, United Arab Emirates; d Department of Cardiology, Mediclinic Parkview Hospital, Dubai, United Arab Emirates; e Department of Endocrinology, Imperial College of London Diabetes Center, Abu Dhabi, United Arab Emirates; f Department of Endocrinology and Lipidology, Dar Al Saha Medical Center, Manama, Bahrain; g Department of Cardiology, Rashid Hospital, Dubai, United Arab Emirates; h Department of Cardiology, Al Sabah Hospital, Kuwait; i Department of Cardiology, Burjeel Royal Hospital, AlAin, United Arab Emirates; j Department of Clinical Lipidology, Sabah Al Ahmad Cardiac Centre, Kuwait City, Kuwait; k Department of Biochemistry, Sultan Qaboos University Hospital, Al-Khod, Muscat, Sultanate of Oman; l Heart Division, Royal Brompton and Harefield Hospitals, Cardiovascular Academic Group, King's College, London, UK; m Center for Molecular Cardiology, University of Zurich, Switzerland

**Keywords:** Lipoprotein(a), Cardiovascular risk, Atherosclerosis, Gulf countries, Risk stratification, PCSK9 targeted therapy, Antisense therapy

## Abstract

**Background:**

Lipoprotein(a) [Lp(a)] is increasingly recognized as an independent and causal risk factor for atherosclerotic cardiovascular (CV) disease including aortic valve stenosis, myocardial infarction, stroke, peripheral arterial disease and CV death. Despite growing global awareness, significant gaps in diagnosis, risk stratification, and management persist in Gulf countries.

**Objective:**

To develop regionally tailored consensus statements on the clinical management of elevated Lp(a) in Gulf countries using a modified Delphi-based methodology.

**Methods:**

A multidisciplinary panel, mostly of Gulf-based experts in cardiology, lipidology, and endocrinology evaluated 64 evidence-based statements across six thematic domains, including pathophysiology, risk assessment, therapeutic strategies, and implementation challenges. Consensus was defined by ≥ 80% agreement, and relevant literature was reviewed to support each statement.

**Results:**

Strong consensus supported the inclusion of Lp(a) in routine CV risk assessment, especially for high-risk individuals. The panel endorsed early detection and aggressive management of modifiable risk factors, including LDL-C, apoB, and non-HDL-C. Areas of partial agreement highlighted practical challenges, including variability in insurance coverage and the need for clearer referral pathways, imaging protocols, and outcome-driven treatment guidance and particularly the current unavailability of specific Lp(a) lowering drugs.

**Conclusion:**

These expert recommendations provide a pragmatic framework for integrating Lp(a) into CV care pathways in Gulf countries. By aligning practice with emerging evidence, the consensus has the potential to shape national guidelines, inform payer policies, promote regional research on the epidemiology of Lp(a), and support equitable access to future Lp(a)-targeted therapies, ultimately improving long-term CV outcomes in the region.

## Highlights

• Lp(a) is a causal and independent risk factor for ASCVD.
• Lp(a) levels are largely genetically determined and stable over life.
• One-time Lp(a) testing recommended, especially in high-risk individuals.
• Elevated Lp(a) should prompt referral for specialized ASCVD risk evaluation.
• Lack of regional data may underestimate risk in Middle Eastern populations.

## Graphical abstract

**Figure:**
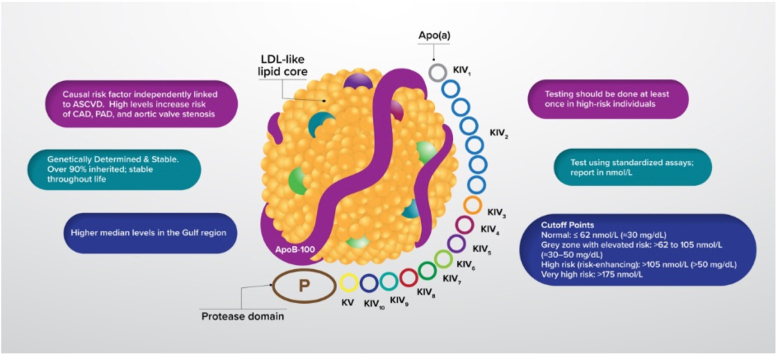

## 1 Introduction

Lipoprotein(a) [Lp(a)] has emerged as a significant and independent CV risk factor, garnering increasing attention in recent years due to its complex structure, genetic basis, and strong association with atherosclerotic cardiovascular disease (ASCVD) and aortic stenosis [1,2]. Structurally, Lp(a) is a plasma lipoprotein particle containing cholesterol, triglycerides, phospholipids, apolipoprotein B [3], and lipoprotein(a) with multiple kringles, sharing some similarities with low-density lipoprotein cholesterol (LDL-C) [4].

Lp(a) is recognized as an independent and primarily genetically determined risk factor for ASCVD including aortic valve stenosis, stroke, and peripheral arterial disease, distinct from traditional lipid parameters [2,5]. Elevated Lp(a) levels contribute to CV risk through multiple mechanisms, including pro-atherogenic, pro-thrombotic, and pro-inflammatory effects [6]. Unlike low-density lipoprotein cholesterol (LDL-C), Lp(a) is not significantly influenced by lifestyle or diet [7], making it a critical yet often overlooked factor in CV risk assessment [8].

In recent years, there has been a resurgence of interest in Lp(a) due to mounting evidence linking elevated levels with adverse CV outcomes, particularly due to Mendelian Randomization studies supporting causality for ASCVD and aortic stenosis [[9], [10], [11]]. This has led to the development of novel therapeutic agents aimed at specifically lowering Lp(a) concentrations [12]. Among these, antisense oligonucleotides (ASO; i.e. Pelacarsen), small interfering ribonucleic acid (siRNA; Olpasiran, Zerlasiran, Lepodiseran), and a novel oral molecule that inhibits the extracellular covalent binding of apo(a) to apoB, thereby reducing Lp(a) assembly (e.g. Muvalaplin), are being actively investigated, showing large reductions in Lp(a) levels and promise of potentially mitigating CV risk [13].

Despite the high burden of cardiovascular disease (CVD), coronary artery disease (CAD), stroke, and cardio-renal-metabolic risk factors in the Middle East [14], there remains a significant gap in the implementation of both, the international and regional guidelines for screening, diagnosis, and management of individuals and patients in the Middle East and particularly the Gulf region [15,16].

**Fig. 1:**
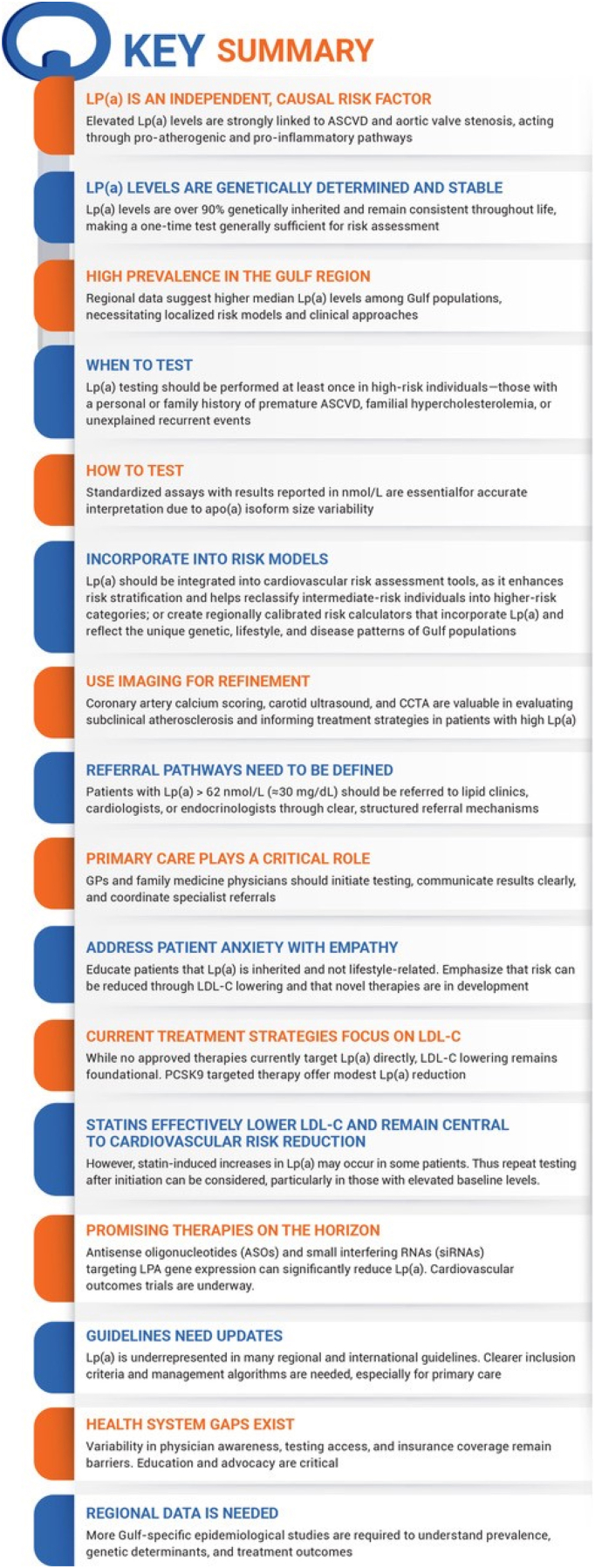
Summary of the key consensus points Lp(a), lipoprotein(a); ASCVD, atherosclerotic cardiovascular disease; Apo(a), apolipoprotein(a); CCTA, Cardiac Computed Tomography Angiography; GP, General Practitioner; LDL-C, low-density lipoprotein cholesterol; PCSK9, Proprotein convertase subtilisin/kexin type 9.

To address this gap, an expert panel of 13 clinicians from the Gulf region convened to develop consensus-based recommendations for the diagnosis and management of Lp(a). Drawing from their collective clinical experience, published literature, and current evidence from international guidelines, the panel aimed to formulate practical, regionally relevant recommendations. A modified Delphi method was employed to develop and validate consensus statements, ensuring the incorporation of expert opinions through a structured and iterative process. A summary of the key consensus points is provided in Fig. 1.

## 2 Methodology

### 2.1 Panel composition

The consensus was formulated through a multi-step process that began with an expert panel meeting comprising thirteen experienced clinicians with extensive publications specializing in endocrinology, lipidology, and cardiology practicing in four Gulf countries, including the United Arab Emirates (UAE), Kuwait, Bahrain, and Oman. The meeting, moderated by an international expert from the United Kingdom, focused on evaluating the clinical significance of Lp(a), reviewing current management strategies, identifying gaps in regional guidelines, and exploring potential recommendations for improving Lp(a) assessment and current treatment in the Gulf Region.

### 2.2 Statement formulation

Prior to the initial meeting, experts completed a structured questionnaire that included both open-ended and closed-ended questions, designed to explore current clinical practices of Lp(a) management, areas of agreement, and topics requiring further discussion, which helped identify areas of agreement and divergence. Responses to this questionnaire informed the agenda and guided the discussions during the meeting, where panelists reviewed the available evidence and engaged in structured discussions to refine key concepts and formulate draft consensus statements. The meeting was transcribed, and draft statements were refined based on the pre-meeting input and live discussions, incorporating current evidence, comprehensive literature reviews, expert opinions, and regional clinical practices. These draft statements were then circulated among the panelists for review and feedback prior to the voting process, to ensure accuracy and alignment with regional clinical needs.

### 2.3 Modified delphi approach

This study employed a modified Delphi approach consisting of three sequential stages: a pre-meeting questionnaire, a structured expert panel discussion, and a final round of anonymous voting. The finalized statements were subjected to a single round of voting via SurveyMonkey. Because preliminary alignment on statement content had already been achieved during these preparatory stages, the voting phase was designed primarily to confirm the level of agreement rather than to iteratively generate statements across multiple rounds; thus, a single round of voting was considered sufficient to determine consensus. Experts rated each statement using five options: strongly agree, agree, neither agree nor disagree, disagree, strongly disagree. For analysis, responses were categorized as follows: “agree” and “strongly agree” were grouped as “A” (agree), “neither agree nor disagree” as “N” (neutral), and “disagree” and “strongly disagree” as “D” (disagree). Voting was conducted anonymously to ensure that panelists could express their opinions independently without influence from other participants. Statements with ≥80% agreement were included in the final consensus, while those with significant disagreement were further analyzed and are discussed in this article. Results were analyzed by an independent researcher to minimize bias and ensure objective interpretation. A total of 64 statements across six thematic domains were formulated (Table 1).

**Table 1:** Experts’ consensus statements on Lipoprotein(a) in the Gulf Region.

No.	Statement	% agreement
**Pathophysiology & Genetics of Lp(a)**		
1	Lp(a) is composed of an ApoB molecule linked to Apo(a)	100
2	Lp(a) is a primarily genetically determined lipoprotein with plasma levels primarily influenced by LPA gene variations	100
3	The number of KIV-2 repeats in Apo(a) determines individual variability in plasma Lp(a) levels and associated cardiovascular risk	100
4	Lp(a) is a proinflammatory and proatherogenic molecule that contributes to cardiovascular disease and aortic stenosis	100
5	Lp(a) levels show minimal variability across an individual's lifetime, unlike LDL-C, which fluctuates based on diet and lifestyle	100
6	Significant ethnic variability exists in Lp(a) levels, with Middle Eastern populations exhibiting higher levels than Western populations	92.31
7	Risk calculators based on Western populations may underestimate cardiovascular risk in Middle Eastern individuals	92.31
8	Mendelian randomization studies have demonstrated that lifelong exposure to higher Lp(a) levels is strongly and causally associated with an increased risk of ASCVD and aortic stenosis	100
9	Very low Lp(a) levels have been associated with an increased risk of diabetes, warranting further research	84.62
**Lp(a) as a Cardiovascular Risk Factor**		
10	Elevated Lp(a) levels are an independent risk factor for ASCVD, aortic stenosis, and venous thromboembolism	84.62
11	Lp(a) is a risk enhancer in cardiovascular risk stratification and should be considered when evaluating ASCVD risk	100
12	A dose-dependent relationship exists between Lp(a) levels and ASCVD events, supporting its role as a continuous and causal risk factor	100
13	Lp(a) is an independent predictor of recurrent CHD in postmenopausal women. The potential role of hormone replacement therapy in reducing Lp(a)-mediated risk in postmenopausal women requires further investigation	84.62
14	Lp(a) contributes to residual cardiovascular risk despite optimal LDL-C control, necessitating separate consideration in risk assessment	100
15	Risk assessment models that incorporate Lp(a) levels improve patient stratification and allow for more precise preventive strategies	100
16	Patients with significantly elevated high Lp(a) levels are at particularly high risk and may require earlier and more intensive intervention	100
**Measurement & Risk Stratification**		
17	Lp(a) should be measured at least once in an individual's lifetime, particularly in high-risk populations, as recommended by the EAS	92.31
18	Lp(a) testing should be prioritized for individuals with a personal or a strong family history of premature ASCVD, recurrent cardiovascular events, familial hypercholesterolemia, or aortic stenosis	92.31
19	Standardized assays for Lp(a) measurement should be established to ensure accuracy, reproducibility, and consistency across different laboratories	100
20	Lp(a) levels should be reported in nmol/L to account for isoform size heterogeneity	100
21	Risk stratification models should incorporate Lp(a) to improve classification of patients into low, moderate, or high-risk categories	100
22	Lp(a) measurement should be used to reclassify patients from moderate to high risk when traditional risk scores underestimate their cardiovascular risk	100
23	Reverse cascade screening should be implemented, where first-degree relatives of individuals with high Lp(a) are also tested	100
24	Lp(a) measurement should be repeated after the initiation of PCSK9 targeted therapy as a decrease in its plasma levels can be expected. Similarly, such measurements should be obtained in statin therapy in patients with elevated levels, as a subset of those experience an increase in Lp(a) levels following statin therapy	100
25	Lp(a) and CRP are markers of residual cardiovascular risk that, while not directly modifiable, provide critical insight into absolute cardiovascular risk. An elevated Lp(a) or CRP level should prompt a more intensive management approach, including stricter blood pressure control, LDL-C lowering, triglyceride management, and glucose optimization	100
26	Residual cardiovascular risk remains a major challenge, particularly in patients with elevated Lp(a). Clinicians should recognize that risk is cumulative, and addressing all modifiable risk factors aggressively is essential, particularly in primary prevention	100
27	The integration of general cardiovascular risk models such as QRISK3 with Lp(a)-specific risk calculators may enhance cardiovascular risk estimation by accounting for Lp(a)-mediated risk and other metabolic parameters. The combined use of these tools can be considered to help assess how much Lp(a) contributes to overall cardiovascular risk and to refine treatment decisions accordingly	92.31
28	QRISK3 may provide additional context for risk estimation in younger patients and those with additional risk factors, while Lp(a)-specific calculators may help contextualize Lp(a)-related residual risk and guide lipid-lowering strategies	92.31
29	Current risk calculators underestimate cardiovascular risk in Middle Eastern populations. We recommend regional validation and adaptation of these tools to better reflect the unique Lp(a) burden and cardiovascular risk profile in Middle Eastern and other underrepresented populations	100
30	The Gulf region should consider developing its own risk assessment model incorporating Lp(a), given its higher prevalence and unique genetic predispositions	84.61
**Guideline & Clinical Practice Gaps**		
31	There is a general lack of awareness among physicians regarding the significance of Lp(a) as a cardiovascular risk factor	92.31
32	Current guidelines inadequately address Lp(a) testing in routine cardiovascular risk assessment	84.62
33	Lp(a) testing rates are low in the Gulf region due to lack of guideline recommendations and physician awareness	92.31
34	Updated international and regional guidelines should incorporate specific recommendations for Lp(a) testing and risk stratification	92.31
35	Physicians across specialties, including general cardiologists, family medicine physicians, and general practitioners, require target education on Lp(a) interpretation and its integration into clinical decision-making	100
36	Insurance coverage for Lp(a) testing and potential treatment remains inconsistent, particularly for primary prevention. Advocacy is needed to improve access	84.62
37	There is an urgent need for structured training programs to educate healthcare professionals on the role of Lp(a) in cardiovascular disease and the clinical utility of emerging therapies	100
38	Comprehensive risk stratification should be performed for patients with elevated Lp(a), integrating traditional cardiovascular risk factors, imaging modalities, and clinical history	100
39	In patients with high Lp(a), imaging techniques such CCTA, including CAC scoring and CIMT assessment can aid in identifying subclinical atherosclerosis and refining risk prediction as a basis of therapeutic decision making	92.31
**Current & Future Therapeutic Strategies**		
40	LDL-C should be aggressively lowered as the primary intervention for patients with elevated Lp(a), as this remains the most effective modifiable risk factor in the absence of direct Lp(a)-lowering therapies	92.31
41	Lipid-lowering strategies should be personalized based on overall cardiovascular risk, with the potential addition of ezetimibe, bempedoic acid, or combination therapy where needed	100
42	PCSK9 targeted therapy should be considered as a second-line therapy to further reduce LDL-C levels and achieve additional reductions in overall cardiovascular risk, particularly in high-risk patients with elevated Lp(a) and residual risk despite maximally tolerated statins and ezetimibe. Their effect on Lp(a) is modest	100
43	Statins, despite their lack of direct effect on Lp(a) levels, should continue to be used in patients with concurrent high LDL-C, ensuring maximally tolerated lipid-lowering therapy	100
44	Patients with recurrent cardiovascular events despite optimal lipid management should be prioritized for future Lp(a)-targeting therapies such as ASO and siRNA once they become available	100
45	A tiered approach to management should be implemented: lifestyle interventions for borderline cases, aggressive LDL-C reduction for high-risk patients, and targeted Lp(a)-lowering therapies once available	92.31
46	In cases of extreme Lp(a) elevation (>200 nmol/L) and very high cardiovascular risk, LDL apheresis may be considered as a temporary intervention while awaiting targeted pharmacological treatments	84.61
47	The psychological burden of high Lp(a) should be addressed through patient education on its genetic basis, risk mitigation strategies, and upcoming treatment advancements	100
**Primary vs. Secondary Prevention Considerations**		
48	Primary prevention should focus on early identification of individuals with high Lp(a) levels and aggressive risk factor modification, including intensive LDL-C lowering, lifestyle interventions, smoking cessation and periodic imaging assessments such as CAC scoring for refined risk prediction	100
49	Secondary prevention should prompt more aggressive lipid-lowering strategies and consideration of PCSK9 targeted therapy	100
50	Referral pathways should be standardized to ensure that individuals with significantly elevated Lp(a) receive timely consultation with lipidologists, cardiologists, and endocrinologists	92.31
51	Imaging-guided strategies, including CAC scoring and vascular ultrasound, should be emphasized to personalize treatment decisions, particularly for patients with borderline risk	84.62
52	Clinical guidelines should be updated to clearly define Lp(a) thresholds for specialist referral, ensuring a streamlined approach to managing high-risk individuals and reducing variability in clinical decision-making	92.31
53	Insurance coverage for PCSK9 targeted therapies should be considered in patients with high Lp(a) and high cardiovascular risk	76.93
54	There is a need for clearer guidelines on Lp(a) management in primary prevention, particularly regarding treatment thresholds	100
55	Elevated Lp(a) is associated with persistent residual cardiovascular risk despite optimal LDL-C lowering	84.62
56	Statins do not lower Lp(a) and may even slightly increase its levels in some patients	100
57	PCSK9 targeted therapies reduce Lp(a) levels by approximately 15-30% but are primarily indicated for LDL-C reduction	92.31
58	Emerging therapies, such as ASO and siRNA, show promise in lowering Lp(a) levels by up to 90-95%, and may significantly impact cardiovascular outcomes	100
59	Until Lp(a)-lowering therapies with clinical outcome data become available, risk reduction strategies should focus on aggressive LDL-C lowering and lifestyle modifications	100
60	Niacin has modest effects on Lp(a) but is limited by poor tolerability	84.62
61	LDL apheresis is an option for patients with extremely high Lp(a) levels, though it is costly and requires frequent sessions	84.62
62	Further studies are needed to assess Lp(a) prevalence and cardiovascular risk impact in the Gulf region	100
63	Patients with metabolic syndrome, diabetes, or chronic kidney disease may have unique risk profiles requiring tailored management strategies for elevated Lp(a)	84.62
64	Consideration should be given to the psychological impact of a high Lp(a) diagnosis, with appropriate counseling on risk management and future therapeutic options	100

Lp(a), lipoprotein(a); ApoB, apolipoprotein B; Apo(a), apolipoprotein(a); KIV-2, kringle IV subtype 2; LDL-C, low-density lipoprotein cholesterol; ASCVD, atherosclerotic cardiovascular disease; CHD, Coronary heart disease; EAS, European Atherosclerosis Society; PCSK9, Proprotein convertase subtilisin/kexin type 9; CRP, C-reactive protein; QRISK3, QRESEARCH risk estimator version 3; CCTA, Cardiac Computed Tomography Angiography; CAC, coronary artery calcium; CIMT, carotid intima-media thickness; ASO, antisense oligonucleotides; siRNA, small interfering ribonucleic acid.

### 2.4 Data collection and analysis

After voting, results were analyzed to assess consensus levels and identify areas of divergence. Statements that did not meet the agreement threshold were reviewed further to explore differing viewpoints. The final consensus document reflects the collective judgment of the expert panel and aims to provide practical guidance on Lp(a) assessment and management in the Gulf region.

## 3 Results

Statements that are fully supported by published scientific evidence are presented as factual summaries of current knowledge, whereas statements informed primarily by expert opinion or regional clinical experience highlight recommendations or considerations where evidence is limited; each statement is discussed in depth in the manuscript to provide context and rationale.

1. Pathophysiology & Genetics of Lp(a)
  1.1. Lipoprotein(a) is composed of an apolipoprotein B (ApoB) molecule linked to apolipoprotein(a) [apo(a)] (*A* = 100%, *N* = 0%, *D* = 0%)
  1.2. Lp(a) is a primarily genetically determined lipoprotein with plasma levels primarily influenced by LPA gene variations (*A* = 100%, *N* = 0%, *D* = 0%)
  1.3. The number of Kringle IV type 2 repeats in Apo(a) determines individual variability in plasma Lp(a) levels and associated cardiovascular risk (*A* = 100%, *N* = 0%, *D* = 0%)
  1.4. Lp(a) is a proinflammatory and proatherogenic molecule that contributes to cardiovascular disease and aortic stenosis (*A* = 100%, *N* = 0%, *D* = 0%)
  1.5. Lp(a) levels show minimal variability across an individual's lifetime, unlike LDL-C, which fluctuates based on diet and lifestyle (*A* = 100%, *N* = 0%, *D* = 0%)
  1.6. Significant ethnic variability exists in Lp(a) levels, with Middle Eastern populations exhibiting higher levels than Western populations (*A* = 92.31%, *N* = 7.69%, *D* = 0%)
  1.7. Risk calculators based on Western populations may underestimate cardiovascular risk in Middle Eastern individuals (*A* = 92.31%, *N* = 7.69%, *D* = 0%)
  1.8. Mendelian randomization studies have demonstrated that lifelong exposure to higher Lp(a) levels is strongly and causally associated with an increased risk of atherosclerotic cardiovascular disease (ASCVD) and aortic stenosis (*A* = 100%, *N* = 0%, *D* = 0%)
  1.9. Very low Lp(a) levels have been associated with an increased risk of diabetes, warranting further research (*A* = 84.62%, *N* = 15.38%, *D* = 0%)

Lp(a) is a distinct lipoprotein particle composed of a lipid-rich core, primarily consisting of cholesteryl esters, and a highly polymorphic glycoprotein, apo(a) [6,17,18]. Apo(a) is covalently linked to apolipoprotein B-100 (ApoB-100) via a single disulfide bond [4], positioned near the binding site of ApoB for the low-density lipoprotein (LDL) receptor [[19], [20], [21]]. Due to its distinct composition, Lp(a) exhibits pro-atherogenic, pro-thrombotic, and pro-inflammatory properties [22], significantly contributing to ASCVD and aortic stenosis [23].

The gene encoding apo(a) (LPA) evolved from the plasminogen gene (PLG) [24], highlighting its evolutionary origin. Despite structural resemblance to plasminogen, including the presence of kringle domains IV and V and a protease domain, apo(a) lacks the functional fibrinolytic activity of plasminogen [25]. Notably, apo(a) contains ten kringle IV subtypes (1–10), while plasminogen only has one [26]. Among these, kringle IV subtype 2 (KIV-2) in apo(a) exhibits significant copy number variation (ranging from 1 to over 40 copies), resulting in variable isoform sizes [3]. In contrast, plasminogen also contains kringles I, II, and III, which are absent in apo(a) [27]. Although both proteins possess a protease domain, only plasminogen's is active, enabling fibrin breakdown [28]. The structural homology between apo(a) and plasminogen allows Lp(a) to interfere with plasminogen activation, potentially impair fibrinolysis, and contribute to endothelial dysfunction [3,[29], [30], [31]]. Mechanistic studies show these effects in vitro and in animal models, but available clinical data do not indicate a significant risk for non-atherosclerotic thrombotic events; nonetheless, they could still contribute to the instability of atherosclerotic plaques [32].

**Fig. 2:**
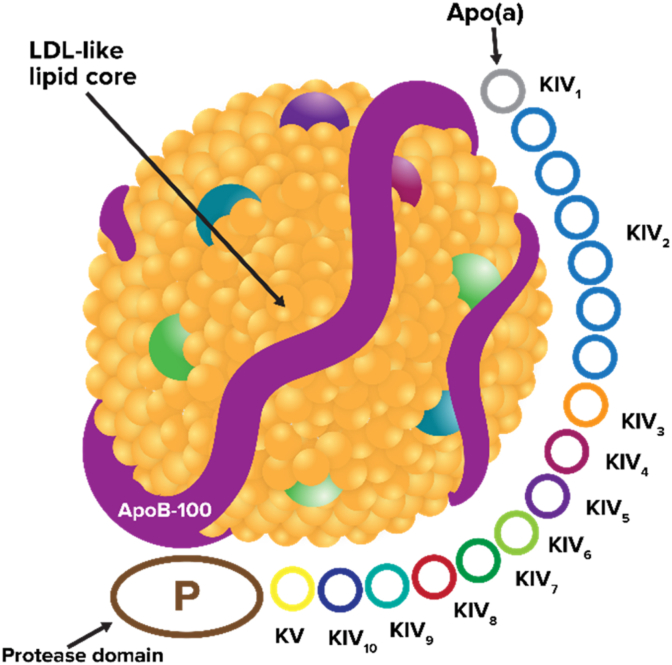
A schematic representation of Lipoprotein(a).

Lp(a) concentration is predominantly genetically determined, with over 90% of its concentration variation attributed to the LPA gene [23]. Two major genetic mechanisms underlie this variation: the number of KIV-2 repeats [33] and several single nucleotide polymorphisms (SNPs) located within and near the LPA locus, which further modulate expression and isoform size even among individuals with the same number of KIV-2 repeats [3] (Fig. 2).

Lp(a) levels exhibit considerable variability among different populations, with substantial inter-individual differences, ranging from nearly undetectable to over 250 nmol/L [34]. This variability is primarily governed by genetic factors, with the apolipoprotein(a) gene (LPA) accounting for nearly all the genetic variation in plasma Lp(a) levels and an estimated 91% of the total variance in plasma Lp(a) concentration [35,36]. The number of KIV-2 repeats alone explains about 69% of the observed variation in individuals of European ancestry [36]. There is an inverse relationship between KIV-2 repeat number and plasma Lp(a) levels; individuals with fewer repeats exhibit higher circulating Lp(a) concentrations and an increased risk of CV events, and vice versa [37,38]. Large-scale Mendelian randomization studies have demonstrated a causal association between lifelong elevated Lp(a) levels and ASCVD, independent of traditional lipid parameters [39]. Beyond copy number variation, SNPs within the LPA gene also play a significant role in modulating Lp(a) levels [40]. For instance, common SNPs such as rs10455872 and rs3798220 are associated with shorter KIV-2 repeat alleles and higher plasma Lp(a) concentrations, collectively explaining up to 36% of the variance in Lp(a) levels in European populations [11]. These variants are strongly linked to elevated CV risk. In contrast, in individuals of African descent, SNPs like rs9457951 account for a smaller proportion of Lp(a) variability due to differences in linkage disequilibrium with KIV-2 repeats [41]. This highlights a more complex and population-specific genetic architecture of the LPA locus.

Substantial ethnic and regional differences in Lp(a) levels have been consistently reported [42]. Populations in the Middle East tend to have higher median Lp(a) concentrations when compared with Western groups [43,44]. Globally, the distribution of Lp(a) levels varies considerably: the lowest values are generally found among East and Southeast Asians, intermediate levels in individuals from Latin America, South Asia, and the Middle East, and the highest levels are typically seen in people of African descent [3]. Research comparing apo(a) isoform sizes across ethnicities revealed that individuals from East and Southeast Asia commonly possess larger isoforms, whereas those from Arab and African populations tend to have smaller ones [43], reinforcing the inverse relationship between apo(a) isoform size and plasma Lp(a) concentration [45]. This association is accompanied by a linear correlation between Lp(a) concentrations and the risk of ASCVD [46].

This variability has important clinical implications, particularly in populations such as those in the Middle East and particularly the Gulf region, where elevated Lp(a) concentrations are common. In these regions, elevated Lp(a) may contribute disproportionately to the high burden of premature CAD. Premature CAD is typically defined by the onset of clinical ASCVD before age 55 in men and 65 in women [47]. Regional studies have consistently shown that patients in the Middle East present with CAD at significantly younger ages compared to Western cohorts, on average, 11-12 years earlier [48,49]. This epidemiologic trend underscores the potential role of genetically determined risk factors like Lp(a) in driving early-onset ASCVD in the region, in populations with a high prevalence of smaller apo(a) isoforms and elevated Lp(a) concentrations. Supporting this, a single-center study from the UAE found that individuals with premature ASCVD were significantly more likely to undergo Lp(a) testing compared to those with non-premature ASCVD, reflecting heightened clinical awareness of Lp(a) as a contributing risk factor in early disease onset [50].

Conventional CV risk calculators, including the Framingham Risk Score, ESC Score, Pooled Cohort Equations (PCE), and ASCVD Risk Estimator Plus, do not incorporate Lp(a) levels and have not been specifically calibrated for Middle Eastern populations [51]. As a result, these tools may systematically underestimate ASCVD risk in individuals from this region, potentially delaying preventive interventions. A comparative study by Alenazi et al. found notable discrepancies in the performance of widely used risk calculators among East Mediterranean and South Asian populations, underscoring the limitations of applying Western-derived tools to non-Western cohorts [52]. Similarly, research by Al-Shamsi et al. demonstrated that existing calculators showed suboptimal predictive accuracy when externally validated in an Emirati population, further emphasizing the need for region-specific models [53]. The lack of region-specific risk models may lead to suboptimal risk stratification and delayed initiation of preventive therapies. Given that Lp(a) levels are primarily genetically determined and remain relatively constant throughout an individual's lifetime [54,55], a single measurement is generally sufficient for long-term CV risk assessment [23]. This stands in contrast to LDL-C, which is mildly responsive to lifestyle and highly responsive to therapeutic interventions and thus requires ongoing monitoring [56].

Emerging evidence suggests an intriguing association between very low Lp(a) levels and a modestly increased risk of type 2 diabetes mellitus (T2DM) [3]. Recent studies have reported a higher incidence of T2DM among individuals with very low Lp(a) concentrations, although the underlying mechanisms remain poorly understood [57,58]. For example, a prospective cohort study of U.S. women showed a higher diabetes risk in individuals within the lowest Lp(a) quintile compared to the highest [58]. A subsequent meta-analysis incorporating multiple studies reported a 38% increased risk in the lowest quintile (typically <5 mg/dL) [23]. However, the absolute risk increase appears to be small, and the clinical significance of this association remains uncertain. This observation may nevertheless be of interest in regions such as the Gulf, where the background prevalence of diabetes is already high [59]. The biological mechanisms driving this association remain poorly understood. Mendelian randomization studies have yielded inconsistent findings, with some suggesting that the association may be influenced more by the genetic characteristics of Lp(a), such as larger apo(a) isoform size [60] or a higher number of KIV-2 repeats [61], rather than the actual Lp(a) concentration itself. This raises the possibility that pharmacological lowering of Lp(a) may not necessarily confer the same metabolic implications as those observed in individuals with genetically determined very low Lp(a) levels. In clinical practice, the implications of these findings are still unclear. While these therapies are being developed primarily to reduce cardiovascular and aortic valvular risk, it remains uncertain whether achieving very low Lp(a) concentrations could influence diabetes risk. However, since clinical trials typically enroll patients with Lp(a) levels exceeding 70 mg/dL, the extent to which novel therapies (e.g., ASO or siRNA agents) may reduce Lp(a) to very low concentrations (<5 mg/dL), and whether such levels would be associated with metabolic effects similar to those suggested by observational studies, remains uncertain [23]. Until more data from ongoing and future randomized controlled trials become available, this potential risk remains theoretical but should be considered when evaluating the overall benefit-risk balance of novel Lp(a)-lowering strategies. Given that diabetes is a polygenic and multifactorial disease [62], further research is warranted to clarify these associations.

In the Gulf and broader Middle East region, the prevalence of type 2 diabetes is among the highest globally, driven by rapid urbanization, lifestyle transitions (e.g. dietary changes and drinking habits among others), physical inactivity and high rates of obesity [63]. Data from population-based studies in the UAE and other Gulf countries underscore the substantial burden of dysglycemia in this region [64]. Given the proximity and demographic overlap with North African populations, and the considerable intra-regional migration and admixture due to substantial population migration [65], ethnic and genetic diversity may influence both Lp(a) distribution and diabetes susceptibility in these populations. However, the potential modifying role of Lp(a) in diabetes risk within these high-prevalence settings remains insufficiently characterized.

The consensus panel shows strong agreement regarding the pathogenic role of Lp(a) and its genetic determinants, with unanimous support for the established understanding of Lp(a)'s composition, stability, and its contribution to CVD. However, some discrepancies were observed regarding the extent of ethnic variability in Lp(a) levels, particularly in Middle Eastern populations. While there is general agreement that Middle Eastern individuals tend to exhibit higher Lp(a) levels compared to Western populations, it should be noted that solid epidemiological evidence for the Middle East is lacking, while the highest levels of Lp(a) in populations of African descent are well documented [44]. This difference may be related to variations in the genetic architecture of the LPA gene across different populations, as well as differences in regional studies. It is also important to recognize that researcher-assigned or genetically inferred ancestry may not always align with self-identified race or ethnicity, particularly in the Middle East, where historical admixture, transregional migration, intermarriage, and trade have created a high degree of genetic diversity and overlap, and individuals may share partial or substantial African ancestry [66]. These factors blur conventional racial or ethnic classifications and complicate efforts to stratify Lp(a)-related CV risk by population. Similarly, while the majority of the panel supports the notion that Western-derived CV risk calculators are likely to underestimate risk in Middle Eastern individuals, a small subset of experts expressed reservations. These discrepancies may stem from differing clinical experiences or perspectives on the potential for adjusting these tools to better reflect regional risk factors. Overall, the panel emphasizes the need for robust, population-based studies in large and representative Middle Eastern and Gulf cohorts to refine risk assessment models, establish region-specific cutoff values, and develop clinical guidelines that are tailored to the unique genetic and epidemiological profile of this population.

2. Lp(a) as a Cardiovascular Risk Factor
  2.1 Elevated Lp(a) levels are an independent risk factor for ASCVD, aortic stenosis, and venous thromboembolism (*A* = 84.62%, *N* = 0%, *D* = 15.38%)
  2.2 Lp(a) is a risk enhancer in cardiovascular risk stratification and should be considered when evaluating ASCVD risk (*A* = 100%, *N* = 0%, *D* = 0%)
  2.3 A dose-dependent relationship exists between Lp(a) levels and ASCVD events, supporting its role as a continuous and causal risk factor (A = 100%, N = 0%, D = 0%)
  2.4 Lp(a) is an independent predictor of recurrent coronary heart disease (CHD) in postmenopausal women. The potential role of hormone replacement therapy in reducing Lp(a)-mediated risk in postmenopausal women requires further investigation (A = 84.62%, N = 15.38%, D = 0%)
  2.5 Lp(a) contributes to residual cardiovascular risk despite optimal LDL-C control, necessitating separate consideration in risk assessment (A = 100%, N = 0%, D = 0%)
  2.6 Risk assessment models that incorporate Lp(a) levels improve patient stratification and allow for more precise preventive strategies (A = 100%, N = 0%, D = 0%)
  2.7 Patients with significantly elevated high Lp(a) levels are at particularly high risk and may require earlier and more intensive intervention (A = 100%, N = 0%, D = 0%)

Lp(a) has been increasingly recognized as an independent and causal risk factor for ASCVD and aortic stenosis [6,67]. Recent evidence indicates that Lp(a) contributes to the pathogenesis of calcific aortic valve disease by promoting a metabolic shift toward PFKFB3-mediated glycolysis in valvular interstitial cells, which sustains inflammatory activity and accelerates disease progression [68]. Large-scale genetic studies have identified LPA variants, particularly rs10455872 and rs3798220 to be strongly associated with elevated Lp(a) levels and increased aortic valve stenosis risk [69], with rs10455872 homozygotes showing a 4.8-fold risk increase in the EPIC-Norfolk study, while individuals in the highest tertile of lipoprotein(a) levels had a 1.6-fold greater risk, reinforcing findings from other population-based cohorts and instrumental variable analyses supporting a causal relationship [70]. In the Middle East and Gulf regions, emerging research underscores the significance of Lp(a) in CV risk assessment. A recent study from the UAE offers important insight into Lp(a) levels among patients in Gulf countries. Conducted at a major quaternary-care hospital, the study reviewed data from 5677 adults tested for Lp(a) between 2017 and 2023. It found that 15.9% of patients had elevated Lp(a) concentrations above 125 nmol/L (∼50 mg/dL). Notably, a third of those with high Lp(a) also had established CV disease, especially CAD [71]. The study also observed that Lp(a) testing was often prompted by prior CV events or a strong family history, thus possibly overstating prevalence in the above-mentioned cohort. [71]. The relationship between Lp(a) concentrations and ASCVD events appears to be linear and dose-dependent in nature [46,72]. Lp(a) has been established as a risk-enhancing factor in CV risk stratification by both American and European guidelines, particularly in individuals with borderline or intermediate ASCVD risk [23,73].

Elevated Lp(a) contributes to residual CV risk despite optimal LDL-C control [74], and independent of traditional risk factors [11]. Incorporating Lp(a) into clinical risk assessment improves patient stratification and supports more tailored preventive interventions by refining CV risk classification [75,76]. The large UK biobank study by Welsh et al. to evaluate the predictive value of Lp(a) for CVD showed that Lp(a) improved CV risk prediction models, particularly in individuals with intermediate baseline risk [77]. Due to the multifaceted pathogenic role of elevated Lp(a), early detection is essential to enable prompt implementation of intensified CV risk management, including lifestyle interventions and, when appropriate, pharmacologic therapy to potentially lower LDL-C to very low levels (i.e. <1.4 mmol/l) and potentially use proprotein convertase subtilisin/kexin type 9 (PCSK9) inhibitor therapies that modestly lower Lp(a) [69,70,78]. Statins remain the cornerstone of LDL-C-lowering therapy, but in some studies, statins have been associated with a small increase in Lp(a) levels, potentially due to enhanced clearance of high-affinity LDL particles, which could leave lower-affinity Lp(a) particles in circulation [32]. This modest variability should be acknowledged in therapeutic planning [79]. While specific guidelines tailored to the Middle Eastern population are still under development, the 2025 Focused Update of the 2019 ESC/EAS Guidelines for the Management of Dyslipidaemias states that, Lp(a) levels above 50 mg/dL (105 nmol/L) should be considered in all adults as a CV risk-enhancing factor, with higher Lp(a) levels associated with a greater increase in risk with recommendation class IIA and evidence B [80], thus supporting a proactive approach in managing high-risk individuals.

Lp(a) has also been identified as an independent predictor of recurrent CHD in postmenopausal women, a population particularly vulnerable to CV events due to hormonal changes during the menopausal transition [81]. Estrogen deficiency associated with menopause is linked to adverse lipid remodeling—including increases in total cholesterol, LDL-C, triglycerides, and in particular Lp(a)—and a reduction in HDL-C, collectively contributing to a more atherogenic lipid profile [81]. While Lp(a) levels are largely genetically determined, several studies have observed an increase in its concentration during and after menopause, suggesting that sexual hormone changes may modulate Lp(a) expression [82]. Indeed, mechanistically, estrogen appears to downregulate LPA gene transcription, while elevated Lp(a) levels promote inflammatory gene expression in vascular tissues, further contributing to ASCVD [83]. Accordingly, women receiving hormone replacement therapy (HRT) tend to have lower Lp(a) levels compared to those who are not on HRT [84]. However, despite these findings, randomized trials such as Heart and Estrogen/progestin Replacement Study (HERS) and the Women's Health Initiative have not demonstrated a net CV benefit from HRT in the general population [85,86]. Instead, they reported increased risks of adverse outcomes such as thromboembolic events, stroke, and breast cancer [87]. Consequently, estrogen therapy is not currently recommended as a targeted treatment for elevated Lp(a), although it may offer selective benefit in high-risk subgroups—a hypothesis that warrants further investigation [88].

Region-specific data from the Middle East and Gulf region are limited [44], but a recent study by Aljawini et al. (2023) evaluated the relationship between Lp(a) levels, menopausal status, and adiposity in Saudi women [82]. Postmenopausal women had significantly higher Lp(a) concentrations than premenopausal and perimenopausal women, reinforcing the hypothesis that estrogen loss may contribute to Lp(a) elevation [82]. These findings align with global data and emphasize the need for further research to explore the CV implications of elevated Lp(a) in postmenopausal women, particularly within the Gulf region, where population-specific lipid patterns may differ. Collectively, the available evidence supports the expert consensus that Lp(a) is an independent predictor of recurrent CHD in postmenopausal women. Although HRT has shown the potential to lower Lp(a), its clinical role in mitigating Lp(a)-mediated CV risk remains uncertain and should be explored further in future studies, particularly those focused on regionally representative cohorts. Moreover, HRT is generally not recommended for ASCVD risk management in women according to the Women's Health Initiative findings, and in fact may increase the risk of venous thromboembolism [89,90]. The lack of unanimous agreement among experts may reflect differing interpretations of the existing evidence on HRT's CV impact, concerns over its safety profile, and the limited availability of region-specific data, which together contribute to uncertainty regarding its utility in managing elevated Lp(a) in postmenopausal women.

The association between elevated Lp(a) levels and venous thromboembolism (VTE) remains an area of active investigation, contributing to the lack of unanimous agreement among experts on this statement. While elevated Lp(a) levels are firmly linked to ASCVD risk and guide risk stratification and clinical management, there is no validated threshold or consistent evidence supporting its use in assessing VTE risk [91,92]. A prospective cohort study by Rodger et al. evaluated whether elevated Lp(a) levels are associated with the risk of recurrent VTE following a first unprovoked event. Among 510 patients followed for a mean of 16.9 months after completing anticoagulation, no significant association was found between Lp(a) concentrations and VTE recurrence, and a threshold of 0.3 g/L) (∼30 mg/dL or ∼72 nmol/L) did not predict recurrent risk. Notably, the study did not identify any predictive Lp(a) threshold, neither in the overall population nor within sex-specific subgroups, highlighting the lack of discriminatory value of Lp(a) in forecasting VTE recurrence [93]. Mechanistically, Lp(a)'s structural similarity to plasminogen has led to hypotheses that it may impair fibrinolysis and promote thrombosis; however, these concepts are largely based on in vitro data and have not been shown to translate into a clinically meaningful risk of non-atherosclerotic thrombosis in humans [23,32]. Observational studies in humans have shown only a modest increase in VTE risk at very high Lp(a) levels, while Mendelian randomization analyses have not confirmed a causal relationship [94]. Thus, Lp(a)'s impact shows to be more confined to atherosclerotic cardiovascular risk rather than venous thromboembolic disease. In the Middle East, where Lp(a)-related research remains limited, the lack of regional data on VTE further complicates consensus. These uncertainties likely account for the differing expert views regarding the inclusion of VTE alongside ASCVD and aortic stenosis in the context of Lp(a)-mediated risk.

3. Measurement & Risk Stratification
  3.1. Lp(a) should be measured at least once in an individual's lifetime, particularly in high-risk populations, as recommended by the European Atherosclerosis Society (EAS) (A = 92.31%, N = 7.69%, D = 0%)
  3.2. Lp(a) testing should be prioritized for individuals with a personal or a strong family history of premature ASCVD, recurrent cardiovascular events, familial hypercholesterolemia, or aortic stenosis (A = 92.31%, N = 0%, D = 7.69%)
  3.3. Standardized assays for Lp(a) measurement should be established to ensure accuracy, reproducibility, and consistency across different laboratories (A = 100%, N = 0%, D = 0%)
  3.4. Lp(a) levels should be reported in nmol/L to account for isoform size heterogeneity (A = 100%, N = 0%, D = 0%)
  3.5. Risk stratification models should incorporate Lp(a) to improve classification of patients into low, moderate, or high-risk categories (A = 100%, N = 0%, D = 0%)
  3.6. Lp(a) measurement should be used to reclassify patients from moderate to high risk when traditional risk scores underestimate their cardiovascular risk (A = 100%, N = 0%, D = 0%)
  3.7. Reverse cascade screening should be implemented, where first-degree relatives of individuals with high Lp(a) are also tested (A = 100%, N = 0%, D = 0%)
  3.8. Lp(a) measurement should be repeated after the initiation of PCSK9 targeted therapy as a decrease in its plasma levels can be expected. Similarly, such measurements should be obtained in statin therapy in patients with elevated levels, as a subset of those experience an increase in Lp(a) levels following statin therapy (A = 100%, N = 0%, D = 0%)
  3.9. Lp(a) and C-reactive protein (CRP) are markers of residual cardiovascular risk that, while not directly modifiable, provide critical insight into absolute cardiovascular risk. An elevated Lp(a) or CRP level should prompt a more intensive management approach, including stricter blood pressure control, LDL-C lowering, triglyceride management, and glucose optimization (A = 100%, N = 0%, D = 0%)
  3.10. Residual cardiovascular risk remains a major challenge, particularly in patients with elevated Lp(a). Clinicians should recognize that risk is cumulative, and addressing all modifiable risk factors aggressively is essential, particularly in primary prevention (A = 100%, N = 0%, D = 0%)
  3.11. The integration of general cardiovascular risk models such as QRISK3 with Lp(a)-specific risk calculators may enhance cardiovascular risk estimation by accounting for Lp(a)-mediated risk and other metabolic parameters. The combined use of these tools can be considered to help assess how much Lp(a) contributes to overall cardiovascular risk and to refine treatment decisions accordingly (A = 92.31%, N = 0%, D = 7.69%)
  3.12. QRISK3 may provide additional context for risk estimation in younger patients and those with additional risk factors, while Lp(a)-specific calculators may help contextualize Lp(a)-related residual risk and guide lipid-lowering strategies (A = 92.31%, N = 7.69%, D = 0%)
  3.13. Current risk calculators underestimate cardiovascular risk in Middle Eastern populations. We recommend regional validation and adaptation of these tools to better reflect the unique Lp(a) burden and cardiovascular risk profile in Middle Eastern and other underrepresented populations (A = 100%, N = 0%, D = 0%)
  3.14. The Gulf region should consider developing its own risk assessment model incorporating Lp(a), given its higher prevalence and unique genetic predispositions (A = 84.61%, N = 7.69%, D = 7.69%)

The European Atherosclerosis Society (EAS) and the European Society of Cardiology (ESC) both advocate measuring Lp(a) at least once in an individual's lifetime, especially in high-risk populations [23,80]. This recommendation reflects the stable, primarily genetically determined nature of Lp(a) concentrations and the strong evidence linking elevated levels to increased ASCVD [6,95]. In addition, the most recent 2026 American College of Cardiology/American Heart Association guidelines clearly endorse universal Lp(a) testing, explicitly recommending that Lp(a) concentration be measured at least once in all adults to improve ASCVD risk assessment [96]. Repeat testing may be warranted in select clinical situations where changes in Lp(a) levels are suspected. However, the consensus among experts was not absolute (Agreement: 92.31%). The slight divergence in opinions may stem from concerns about the cost-effectiveness of universal screening, potential psychological impacts of identifying elevated Lp(a) without available targeted therapies, and variability in healthcare infrastructure across regions. Prioritizing Lp(a) testing for individuals with a personal or family history of premature ASCVD, recurrent CV events, familial hypercholesterolemia, or aortic stenosis is widely endorsed [69,72,[97], [98], [99], [100]]. These populations are at a heightened risk, and Lp(a) measurement can enhance risk stratification and guide clinical decision making [101]. The absence of unanimous agreement on statement 3.2 does not reflect disagreement with the recommendation itself; rather, it stems from diverging opinions on universal testing, regardless of baseline risk. This perspective, while supportive of the importance of Lp(a) testing, extended beyond the specific scope of prioritizing high-risk groups and highlights an even stronger endorsement of Lp(a) as a key component of CV risk assessment. While current guidelines stop short of recommending universal screening due to the absence of specific outcome-modifying therapies, the low cost and clinical utility of the test suggest minimal downside [88]. Expert consensus was unanimous (100%) in supporting the integration of Lp(a) into risk models and its utility in reclassifying risk. Indeed, elevated Lp(a) is far more prevalent than familial hypercholesterolemia and, as a genetically determined dyslipidemia, represents the most common worldwide [102].

As targeted therapies continue to emerge, the case for routine, population-wide Lp(a) testing may warrant reconsideration in future clinical frameworks. Numerous studies have shown that adding Lp(a) improves risk estimation beyond traditional factors like LDL-C, diabetes, or blood pressure, and aids reclassification, particularly in intermediate-risk individuals [74,80,[103], [104], [105]].

The accurate measurement of Lp(a) is technically complex due to its structural heterogeneity, driven by variations in the number of KIV-2 repeats in apo(a), as mentioned previously [106]. These variations influence not only Lp(a) concentration but also the binding affinity of immunoassays, resulting in significant inter-laboratory variability [34]. A major challenge lies in the development of antibodies that can reliably detect apo(a) across its diverse isoforms without cross-reacting with structurally similar proteins like plasminogen or apoB [26]. The size heterogeneity of apo(a) alters the number of antigenic determinants per particle, leading to isoform-dependent inaccuracies that compromise assay calibration and clinical interpretation [26]. Consequently, the use of standardized assays and reporting in molar units (nmol/L) has been strongly recommended by to reflect the number of circulating particles and not the variable mass of apo(a) or the lipid component [88], thus improving consistency and enabling meaningful comparison across studies, [107,108]. Although regional data remain limited, the relatively high prevalence of elevated Lp(a) in Gulf populations [71]suggests that incorporating Lp(a) into local risk algorithms could significantly enhance preventive strategies.

Cascade screening of first-degree relatives of individuals with elevated Lp(a) is a highly effective strategy, given that Lp(a) levels are over 90% genetically determined [109]. Studies have shown that testing relatives of probands with both familial hypercholesterolemia (FH) and high Lp(a) identifies one new case for every 2.4 screened, while screening from probands with only FH yields one case per 5.8 screened [55]. These findings support the integration of Lp(a) testing into existing FH cascade screening programs [109]. The coexistence of FH and elevated Lp(a) is associated with markedly increased ASCVD risk, and early identification through family-based screening, including in pediatric populations, can enable timely risk mitigation, as demonstrated by the SAFEHEART trial in 2019 [110]. The 2025 ESC/EAS guidelines define Lp(a) concentrations ≥105 nmol/L (≈50 mg/dL) as elevated, and several studies have reported favorable numbers needed to screen when using thresholds in this range, particularly at levels above 125 nmol/L [71,80,111]. To note that there is no universally accepted conversion factor between mg/dL and nmol/L for Lp(a), as the mass of Lp(a) particles varies according to apo(a) isoform size, resulting in assay-dependent differences. Consequently, unit conversion remains approximate, with factors in the literature typically ranging from 2.0 to 2.5 [112]. Some large population-based studies have standardized conversions by dividing mg/dL values by 2.15 [46,113] or by applying the formula Lp(a) (nmol/L) = [2.18 × Lp(a) (mg/dL)] − 3.83, as reported in the Mass General Brigham Lp(a) Registry and analyses by Berman et al. [114,115]. Given the strong heritability and high diagnostic yield, the expert panel unanimously endorsed reverse cascade screening as a practical and evidence-based approach to identify at-risk individuals early and improve CV prevention efforts.

Pharmacologic agents such as PCSK9 targeted therapies (e.g., alirocumab, evolocumab, inclisiran) have been shown to reduce Lp(a) levels by approximately 20–30%, although the clinical significance of this reduction is still being evaluated [[116], [117], [118]]. Although these agents are not specifically approved for the treatment of elevated Lp(a), their use has been associated with a reduction in CV events, particularly in patients with established ASCVD [3,119]. The clinical benefit appears to be driven by both LDL-C lowering and modest reductions in Lp(a), though the independent contribution of Lp(a) lowering remains under investigation [119]. Large-scale CV outcome trials have been instrumental in establishing the clinical efficacy and safety of PCSK9 targeted therapy. For example, The FOURIER trial demonstrated that evolocumab lowered Lp(a) levels by a median of 26.9% and was associated with a reduction in CV events [120]. A pooled analysis of the ORION-10 and ORION-11 trials showed that twice-yearly inclisiran reduced LDL-C by ∼52% over 18 months in patients with ASCVD, with 88% achieving LDL-C <55 mg/dL, and was well tolerated across all subgroups. In the total ASCVD population, median Lp(a) fell by 17.9% with inclisiran vs a 3.3% rise with placebo [121]. Conversely, statins have been reported to paradoxically increase Lp(a) levels in a subset of patients [79], prompting some experts to recommend repeat Lp(a) measurement after initiating statin therapy to reassess residual risk. For example, a prospective study involving 488 patients with acute coronary syndrome (ACS) who underwent percutaneous coronary intervention (PCI) and received statin-based therapy found that, on average, Lp(a) levels rose by approximately 19.3% within one month of statin initiation. Over a three-year follow-up, those who experienced a marked increase in Lp(a) had a significantly higher incidence of MACE, including myocardial infarction, stroke, and unplanned revascularization, compared to those whose Lp(a) remained stable [122]. However, these findings are not universally observed. A systematic review and meta-analysis of multiple trials concluded that statin therapy does not cause clinically meaningful changes in Lp(a) levels compared with placebo, and therefore, statins are unlikely to substantially alter Lp(a)-associated cardiovascular risk in most patients [123]. Taken together, while some individuals may experience notable increases in Lp(a), other evidence indicates that statins generally do not affect Lp(a) to a clinically relevant degree [3,124]. Given this variability, the expert panel recommended reassessing Lp(a) after initiation of statin or PCSK9 therapy in selected patients to better evaluate residual risk and guide management.

**Fig. 3:**
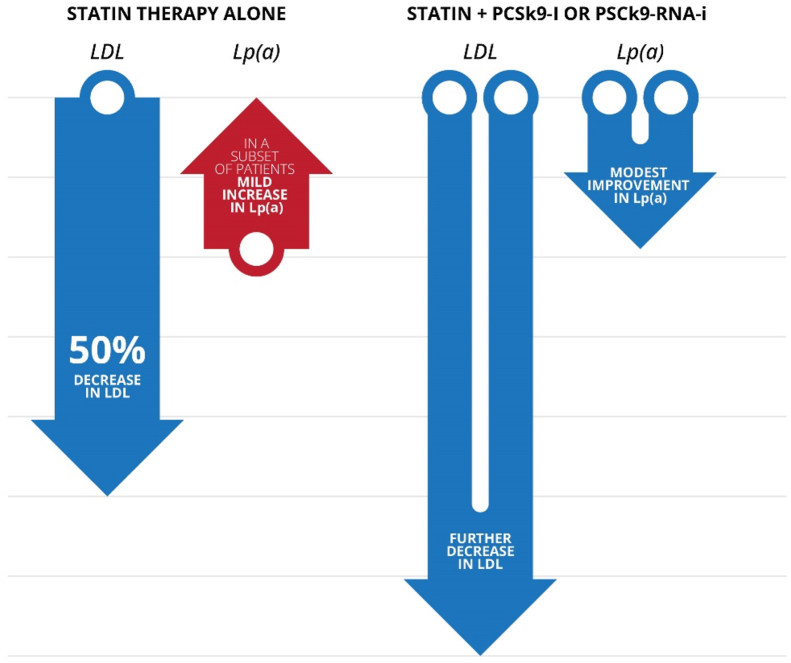
Schematic representation of the complementary effects of adding PCSK9 inhibition to statin therapy, resulting in enhanced LDL-C reduction and a modest lowering of lp(a).

In this context, following statin therapy, LDL-C can be considerably reduced while Lp(a) may rise modestly in some patients; the addition of PCSK9-targeted therapies to statins, particularly in those who fail to achieve LDL-C targets on maximally tolerated statin therapy or who demonstrate statin-induced Lp(a) increases, enables even further LDL-C lowering while also achieving modest reductions in Lp(a), as demonstrated in the ORION trials. This integrated approach highlights the value of combination therapy in addressing residual cardiovascular risk associated with elevated Lp(a). This concept is summarized in Fig. 3.

Elevated Lp(a) and high-sensitivity C-reactive protein (CRP) serve as markers of residual CV risk, providing insights into absolute risk levels [125,126]. Therefore, their presence should prompt a more aggressive approach to managing modifiable risk factors, including LDL-C lowering, blood pressure control, triglyceride reduction, and glucose optimization. Residual CV risk remains a major challenge, particularly in individuals with elevated Lp(a), reinforcing the importance of a comprehensive risk-reduction strategy [74,113].

Accurate CV risk prediction is critical for guiding prevention strategies, particularly in populations with a high burden of non-traditional risk factors such as elevated Lp(a) [110]. However, most commonly used risk prediction tools, including the Framingham Risk Score and European SCORE, were developed in Western populations and consistently underestimate ASCVD risk in Middle Eastern cohorts [127,128]. This discrepancy could be attributed to regional differences in genetic predispositions, lipid profiles, age of disease onset, and the prevalence of emerging risk factors like Lp(a). CVD poses a significant health challenge in Gulf countries, with rates that are notably higher than global averages. For instance, in 2021, the UAE reported the highest age-standardized prevalence rate of CVD in the North Africa and Middle East region [129]. There is a notable lack of large-scale epidemiological studies on Lp(a) in the Gulf region, limiting the ability to accurately assess its population-level distribution and clinical impact. As outlined earlier, a recent study from the UAE provided valuable regional insight by analyzing over 5600 Lp(a) test results from a quaternary-care center found that 15.9% of patients had elevated Lp(a) levels, and a substantial proportion of those individuals had underlying CV conditions such as CAD, heart failure, or stroke [71]. Meanwhile, a prospective study in Oman examining Lp(a) levels in patients with premature acute coronary syndrome is underway, but its findings have not yet been published [130]. These early efforts highlight the urgent need for broader epidemiological research that can underpin Gulf-specific guidelines for Lp(a) detection and clinical intervention.

Although few validated, population-specific CV risk calculators exist for the Gulf region, the Abu Dhabi Cardiovascular Risk Index (ADCRI) represents a noteworthy step toward locally tailored risk prediction [131]. However, awareness of the tool among clinicians outside the UAE may be limited, and despite its validation within its development cohort, it has not yet achieved widespread regional adoption. which could explain why QRISK3 has gained broader traction as an interim option. In the absence of broadly validated Gulf-specific tools, some clinicians have pragmatically utilized established international risk models for risk estimation. Among these, QRISK3 has been discussed in clinical practice as a potentially informative model because it incorporates a broader range of variables than many traditional risk calculators, including ethnicity, comorbidities, and socioeconomic determinants (age, systolic blood pressure, smoking status, cholesterol levels, body mass index, ethnicity, socioeconomic status, family history, chronic kidney disease, rheumatoid arthritis, atrial fibrillation, diabetes mellitus, and antihypertensive treatment) [132]. Additionally, QRISK3 can be applied to individuals as young as 25 years of age, an important consideration given the earlier onset of ASCVD observed in the region, and has been specifically highlighted in the 2022 Middle East Dyslipidemia Guidelines [133,134]. However, QRISK3 has not been formally validated in Gulf populations, and therefore its use should be considered exploratory rather than definitive. Although the model does not include Lp(a) as a formal variable, its flexibility and broader inclusion of ethnicity and comorbidities may offer a conceptual framework for risk estimation until regionally calibrated tools become available. The “Lp(a) risk calculator” may serve as a valuable complementary approach to quantify residual risk and to support decisions around early intervention, particularly in individuals with a personal or family history of premature ASCVD or recurrent events despite optimal LDL-C control [72,[135], [136], [137]]. However, this calculator was developed using data primarily from Western biobanks and may carry population-specific biases, limiting their applicability to Middle Eastern populations without regional validation [136]. Although 92.31% of experts supported the combined use of general risk models such as QRISK3 and Lp(a)-specific calculators in tandem, the remaining divergence did not reflect disagreement with their clinical utility, but rather a note of caution regarding the strength of current evidence. The lack of consensus around CV risk scoring systems in the Gulf region complicates efforts to systematically incorporate Lp(a), particularly when existing Lp(a)-focused calculators are derived from specific cohorts such as the UK Biobank. Without agreement on a unified regional risk framework, the addition of Lp(a) may risk further confusion rather than improving clinical clarity. Specifically, some experts emphasized the need to await outcome trials from emerging Lp(a)-lowering therapies to fully understand the risk-benefit balance before formalizing widespread integration into routine risk algorithms [138]. Nonetheless, the panel unanimously agreed (100%) that existing tools underestimate ASCVD risk in Middle Eastern populations and require urgent regional adaptation. The proposal to develop a Gulf-specific risk calculator that incorporates Lp(a) received strong but not universal support (84.61%), with some hesitancy rooted in the logistical demands of establishing large-scale validation cohorts. Still, the elevated Lp(a) prevalence and earlier onset of ASCVD in Gulf populations provide a compelling rationale for developing and implementing more locally calibrated tools. Any such tool should incorporate Lp(a) as a central variable to improve the identification of high-risk individuals who might otherwise go unrecognized in existing Western-derived calculators.

4. Guideline & Clinical Practice Gaps
  4.1. There is a general lack of awareness among physicians regarding the significance of Lp(a) as a cardiovascular risk factor (A = 92.31%, N = 0%, D = 7.69%)
  4.2. Current guidelines inadequately address Lp(a) testing in routine cardiovascular risk assessment (A = 84.62%, N = 15.38%, D = 0%)
  4.3. Lp(a) testing rates are low in the Gulf region due to lack of guideline recommendations and physician awareness (A = 92.31%, N = 7.69%, D = 0%)
  4.4. Updated international and regional guidelines should incorporate specific recommendations for Lp(a) testing and risk stratification (A = 92.31%, N = 7.69%, D = 0%)
  4.5. Physicians across specialties, including general cardiologists, family medicine physicians, and GPs, require target education on Lp(a) interpretation and its integration into clinical decision-making (A = 100%, N = 0%, D = 0%)
  4.6. Insurance coverage for Lp(a) testing and potential treatment remains inconsistent, particularly for primary prevention. Advocacy is needed to improve access (A = 84.62%, N = 15.38%, D = 0%)
  4.7. There is an urgent need for structured training programs to educate healthcare professionals on the role of Lp(a) in cardiovascular disease and the clinical utility of emerging therapies (A = 100%, N = 0%, D = 0%)
  4.8. Comprehensive risk stratification should be performed for patients with elevated Lp(a), integrating traditional cardiovascular risk factors, imaging modalities, and clinical history (A = 100%, N = 0%, D = 0%)
  4.9. In patients with high Lp(a), imaging techniques such as coronary artery CT angiography, including coronary artery calcium (CAC) scoring and carotid intima-media thickness (CIMT) assessment can aid in identifying subclinical atherosclerosis and refining risk prediction as a basis of therapeutic decision making (A = 92.31%, N = 7.69%, D = 0%)

Elevated Lp(a) is a primarily genetically determined form of dyslipidemia (i.e. present since birth) with a prevalence in the general population that is significantly higher than familial hypercholesterolemia and contributes to lifetime risk. Given its high frequency, there is a critical need to increase awareness and involvement of primary care physicians, general practitioners, and other specialties in its identification and management. Despite growing evidence supporting Lp(a) as a causal and independent risk factor for ASCVD and calcific aortic valve disease, awareness among healthcare professionals remains limited. Multiple observational studies have highlighted a pervasive gap in physician knowledge regarding the role of Lp(a) in CV risk, and that Lp(a) is underutilized in clinical practice, even among high-risk populations [25,139]. A recent study by Bhatia et al. examining testing patterns in a large U.S. academic health system found that Lp(a) was infrequently measured, including in patients with established CVD, suggesting persistent gaps in physician awareness or prioritization [140]. A similar trend has been observed in the Middle East, where testing rates remain low and are often restricted to lipid specialists or tertiary care settings [50,71]. In this context, 92.31% of experts in the current consensus agreed that a general lack of physician awareness is a key barrier to broader implementation of Lp(a) testing. The single dissenting vote likely reflects practice within a specialized or referral-based center where Lp(a) awareness and testing are more routinely integrated, but does not represent prevailing trends across the broader healthcare landscape.

Although the most recent 2025 ESC/EAS guidelines and the National Lipid Association (NLA) recommend measuring Lp(a) at least once in a person's lifetime [23,141], and the ESC/EAS Guidelines further recommend to consider Lp(a) levels above 50 mg/dL or 105 nmol/L in all adults as a CV risk-enhancing factor [80], other major international and regional CV guidelines have yet to incorporate Lp(a) testing into routine risk assessment protocols. For instance, the 2019 ACC/AHA Guidelines on Primary Prevention of Cardiovascular Disease acknowledged Lp(a) as a “risk-enhancing factor” but did not provide specific guidelines for diagnosis and management strategies [73]. However, more recent updates from the ACC/AHA mark a significant shift in approach, with the 2026 guidelines now endorsing routine Lp(a) assessment by recommending at least one lifetime measurement in all adults as part of ASCVD risk evaluation [96]. Additionally, Lp(a) is often absent from widely used clinical pathways, particularly in primary care settings, and measurement is not routinely included in standard lipid panels. In this consensus, 84.62% of experts agreed that current guidelines inadequately address Lp(a). The dissenting vote emphasized the need to await outcome trials with Lp(a)-lowering therapies to better understand the balance of risk and benefit before integrating Lp(a) testing more broadly into guidelines. Notably, the Lp(a) HORIZON trial, a randomized, controlled Phase 3 CV outcomes study evaluating the efficacy of Pelacarsen in patients with elevated Lp(a) levels, is currently underway and expected to present results at the ESC Congress in Munich, Germany, in 2026 [142,143]. The outcomes of this and similar trials are expected to inform future guideline recommendations regarding Lp(a) measurement and management.

Despite robust evidence linking Lp(a) to ASCVD, aortic stenosis, and vascular disease, real-world testing rates remain remarkably low across global and regional health systems. In a large observational study across 6 University of California academic medical centers, Lp(a) was tested in only 0.3% of more than 5.5 million adults evaluated between 2012 and 2021 [140]. Similarly, a recent study from the Mayo Clinic, which analyzed over 250,000 individuals who had lipid panels ordered in a single year, found that only 0.15% underwent Lp(a) testing within the same year, and fewer than 1% had ever been tested historically [144]. Notably, Lp(a) testing occurred primarily in secondary prevention settings, and even when elevated Lp(a) levels were identified, they rarely translated into changes in statin or aspirin therapy, underscoring a disconnect between diagnosis and intervention [144]. In spite of the recognition of the unusually high prevalence of premature CAD, in the Gulf region, the trend is no different. The previously mentioned 2024 study from a quaternary care center in the UAE reported that only 0.95% (5677) of nearly 595,658 patients had their Lp(a) levels measured [71]. These data reflect a significant underutilization of Lp(a) testing in both primary and secondary prevention contexts, likely driven by a combination of low physician awareness, lack of standardized clinical protocols, and limited guideline adoption. In the present modified Delphi consensus, 92.31% of experts agreed that testing rates in the Gulf remain unacceptably low. The single neutral vote was not based on disagreement with the problem itself, but rather stemmed from concerns about limited physician familiarity with interpreting elevated Lp(a) results and uncertainty regarding appropriate management strategies in the absence of targeted therapies. Collectively, these findings call for targeted educational initiatives, expanded guideline implementation, and improved access through both health policy and payer-level advocacy.

There is broad consensus that education on Lp(a) should extend beyond lipidologists to general cardiologists, internists, endocrinologists, family physicians and general practitioners. The unanimous expert agreement (100%) reflects the urgent need for targeted, specialty-inclusive educational initiatives. Structured training programs for healthcare professionals are essential to bridge knowledge gaps and facilitate integration into routine care. These programs should cover the interpretation of Lp(a) levels and cutoff points (which should be standardized), selection of candidates for testing, and understanding of therapeutic implications. The unanimous agreement (100%) from experts reflects the pressing need to institutionalize such education in cardiology, endocrinology, and primary care curricula.

In some countries, insurance reimbursement for Lp(a) testing remains inconsistent, particularly in the context of primary prevention, and this variability has been identified as a key barrier to broader clinical adoption [138]. Analysis of large health insurance claims databases, such as those by Stürzebecher et al. have shown that testing is underutilized even among individuals with full reimbursement [145]. While some insurers cover Lp(a) testing when ordered by specialists or within academic centers, coverage is frequently denied in general practice settings or when testing is ordered for preventive purposes, limiting accessibility [141]. This inconsistency is further compounded by regional differences in healthcare systems and payer policies. As highlighted in the NLA's recent update, heterogeneity exists not only in testing rates but also in insurance coverage across institutions and patient populations [88]. Importantly, concerns about insurance coverage have been identified as a contributing factor to clinician hesitation in ordering the test [140]. As noted in a large academic health system analysis, limited reimbursement discourages routine testing, particularly in general practice where guidelines may be less explicit and payer restrictions more stringent [140]. In the current modified Delphi panel, 84.62% of experts agreed that insurance coverage for Lp(a) testing remains insufficient. The few neutral responses likely reflect variability in local payer practices—where reimbursement may be available in select institutions or for patients with advanced CV disease. Also, many health systems and payers remain hesitant to support testing in the absence of actionable outcomes. Taken together, these insights underscore the need for advocacy and policy reform to standardize coverage, reduce inequities in access, and prepare healthcare systems for the integration of future Lp(a)-targeted therapies.

Patients with elevated Lp(a) often experience residual CV risk despite achieving LDL-C control [146]. This persistent risk underscores the importance of comprehensive risk stratification, which includes evaluating traditional risk factors, clinical history, and advanced imaging modalities [137]. Imaging tools such as coronary artery calcium (CAC) scoring, coronary computed tomography angiography (CCTA), and carotid intima-media thickness (CIMT) assessment are instrumental in detecting subclinical atherosclerosis, and refining risk estimates, particularly in individuals with intermediate risk or discordant clinical profiles[[147], [148], [149]]. CAC scoring quantifies calcified plaque in the coronary arteries and has been shown to enhance ASCVD risk classification beyond conventional factors. In a study by Mehta et al. individuals with elevated Lp(a) and higher CAC scores exhibited significantly greater risk for CV events than those with low CAC scores, even when other risk factors were well-controlled [150]. In this modified Delphi consensus, 92.31% of experts endorsed the use of such imaging modalities in patients with elevated Lp(a) to guide therapeutic decisions. The single neutral vote likely reflects concerns regarding the practicality of widespread implementation—such as cost, availability of imaging infrastructure, and the need for region-specific protocols or reimbursement frameworks. Nonetheless, the near-unanimous agreement highlights strong support for a precision-medicine approach that integrates imaging with biomarker data to optimize risk assessment and treatment planning in Lp(a)-driven CV disease.

While ASCVD remains a leading cause of morbidity and mortality in the Gulf, there is a lack of comprehensive survey data evaluating clinical practice patterns and gaps, particularly regarding emerging risk markers such as Lp(a). A systematic review by Koornneef et al. found that relatively few studies in the GCC region have focused on clinical practice guidelines (CPGs), especially outside the context of lifestyle diseases like diabetes and CV conditions. The review also highlighted that while interest in CPGs is growing, the quality of research methodologies needs improvement, and greater attention is required for the development, implementation, and evaluation of guidelines tailored to the region [151]. Notably, no studies have systematically assessed how Lp(a) is being used or understood in clinical practice across Gulf countries. This consensus therefore calls for targeted data collection efforts, including clinician surveys and system-level assessments, to map current practice, identify implementation barriers, and inform policy development. Generating such data is essential for aligning regional care with international standards and supporting the integration of Lp(a)-related innovations into routine practice.

5. Current & Future Therapeutic Strategies
  5.1. LDL-C should be aggressively lowered as the primary intervention for patients with elevated Lp(a), as this remains the most effective modifiable risk factor in the absence of direct Lp(a)-lowering therapies (A = 92.31%, N = 7.69%, D = 0%)
  5.2. Lipid-lowering strategies should be personalized based on overall cardiovascular risk, with the potential addition of ezetimibe, bempedoic acid, or combination therapy where needed (A = 100%, N = 0%, D = 0%)
  5.3. PCSK9 targeted therapy should be considered as a second-line therapy to further reduce LDL-C levels and achieve additional reductions in overall cardiovascular risk, particularly in high-risk patients with elevated Lp(a) and residual risk despite maximally tolerated statins and ezetimibe. Their effect on Lp(a) is modest (A = 100%, N = 0%, D = 0%)
  5.4. Statins, despite their lack of direct effect on Lp(a) levels, should continue to be used in patients with concurrent high LDL-C, ensuring maximally tolerated lipid-lowering therapy (A = 100%, N = 0%, D = 0%)
  5.5. Patients with recurrent cardiovascular events despite optimal lipid management should be prioritized for future Lp(a)-targeting therapies such as antisense oligonucleotides (ASO) and small interfering RNA (siRNA) once they become available (A = 100%, N = 0%, D = 0%)
  5.6. A tiered approach to management should be implemented: lifestyle interventions for borderline cases, aggressive LDL-C reduction for high-risk patients, and targeted Lp(a)-lowering therapies once available (A = 92.31%, N = 7.69%, D = 0%)
  5.7. In cases of extreme Lp(a) elevation (>200 nmol/L) and very high cardiovascular risk, LDL apheresis may be considered as a temporary intervention while awaiting targeted pharmacological treatments (A = 84.61%, N = 7.69%, D = 7.69%)
  5.8. The psychological burden of high Lp(a) should be addressed through patient education on its genetic basis, risk mitigation strategies, and upcoming treatment advancements (A = 100%, N = 0%, D = 0%)

Elevated Lp(a) is a well-established independent risk factor for ASCVD, yet no currently approved therapies directly target Lp(a) levels [152]. In this context, aggressive LDL-C reduction remains the cornerstone of CV risk mitigation [80,153], supported by strong evidence from trials like Vytorin Efficacy International Trial (IMPROVE-IT) and FOURIER [154,155]. Accordingly, 92.31% of experts agreed that LDL-C should be aggressively targeted in patients with high Lp(a), though some noted that all modifiable CV risk factors (not just LDL-C) should be comprehensively addressed to reduce overall risk [72]. These findings demonstrate the independent and additive nature of Lp(a) and LDL-C in contributing to ASCVD risk, and emphasize that LDL-C lowering alone does not fully offset the risk mediated by elevated Lp(a) [146]. While lifestyle interventions have negligible impact on Lp(a) concentrations, they remain essential in mitigating overall CV risk, although are often difficult to implement over prolonged periods of time. Evidence from the EPIC-Norfolk prospective cohort study demonstrated that individuals with high Lp(a) levels who maintained ideal CV health, defined by optimal metrics for body weight, blood pressure, lipids, glucose, physical activity, diet, and smoking status, experienced significantly fewer major adverse cardiovascular events (MACE) compared to those with poor CV health [70].

There was unanimous consensus (100%) supporting a personalized, risk-based approach to lipid-lowering therapy. Statins remain the foundation of lipid-lowering therapy, particularly in patients with concurrent elevated LDL-C [124]. Despite their potential to modestly increase Lp(a) levels in some individuals as mentioned earlier [79], their established benefit in reducing ASCVD events strongly outweighs this concern. This consensus (100%) reflects alignment with current guidelines recommending maximally tolerated statins in nearly all high-risk patients [6,156]. In addition to high-intensity statins, non-statin agents such as ezetimibe and bempedoic acid can offer incremental LDL-C reduction with favorable safety profiles [15]. The CLEAR Outcomes trial demonstrated the CV benefit of bempedoic acid in statin-intolerant patients [157].

All experts (100%) endorsed PCSK9 targeted therapies as appropriate second-line treatment for high-risk patients. As detailed earlier, these agents significantly lower LDL-C and modestly reduce Lp(a) levels by up to 30% [118,158]. Multiple clinic trials have demonstrated their CV benefits: the ODYSSEY OUTCOMES trial showed that alirocumab reduced MACE in patients with recent acute coronary syndrome who continued to have elevated atherogenic lipoproteins despite maximal statin therapy [159,160], while the FOURIER trial demonstrated greater absolute benefit from evolocumab in those with elevated Lp(a) [161]. Inclisiran, a siRNA that inhibits hepatic synthesis of PCSK9, has demonstrated robust lipid-lowering effects of up to 50% in the ORION clinical trial program, particularly ORION-9, ORION-10, and ORION-11 trials [162,163]. As a key secondary outcome, these studies also reported modest but consistent reductions in lipoprotein(a) levels: placebo-corrected percentage decrease of −25.6% in ORION-10 and -18.6% in ORION-11 [164]. While the Lp(a)-lowering effect is secondary, the durable LDL-C reduction justifies their inclusion in the treatment paradigm, and adds to inclisiran's value in managing residual risk. The CV outcomes associated with inclisiran are still under investigation. A pooled patient-level analysis of ORION-9, -10, and −11 trials showed that adding inclisiran to existing lipid-lowering therapy was linked to a 26% reduction in MACE, along with favorable trends in reducing both fatal and non-fatal myocardial infarction compared to placebo [165]. However, conclusive evidence awaits the results of two large ongoing trials: ORION-4 CV outcome trial, which is evaluating the efficacy of inclisiran in reducing MACE among approximately 15,000 patients with established ASCVD, and VICTORION-2 PREVENT, which is assessing the role of inclisiran in the secondary prevention of MACE in 17,000 participants with established CVD [166]. The results of these studies will provide critical insights into the long-term CV benefits of inclisiran therapy.

Notably, emerging evidence from the Phase 3 CORALreef AddOn trial highlights a clear divergence in the effects of PCSK9-targeting versus non-PCSK9 therapies on Lp(a). In this randomized study of patients receiving background statin therapy, the oral PCSK9 inhibitor enlicitide achieved a median reduction in Lp(a) of −26.2% at day 56, whereas non-PCSK9 therapies demonstrated neutral or unfavorable effects, with increases observed in the bempedoic acid (+8.1%) and bempedoic acid–ezetimibe combination (+10.4%) groups, and no meaningful change with ezetimibe alone [167]. These findings further reinforce the distinct role of PCSK9 pathway modulation in reducing Lp(a), in contrast to conventional lipid-lowering therapies, which may fail to address, or potentially exacerbate, Lp(a)-mediated residual cardiovascular risk.

A clear consensus (100%) was reached regarding the prioritization of patients with elevated Lp(a) for novel targeted therapies once they become available. Several advanced therapies are in late-stage development and show promising efficacy in substantially lowering Lp(a) levels through targeted gene-silencing approaches. Pelacarsen, an ASO, inhibits apo(a) production by binding to LPA mRNA in hepatocyte nuclei, thereby preventing translation [23]. It is administered via once-monthly subcutaneous injection and has demonstrated up to 80% Lp(a) reduction in early-phase studies, with over 80% of patients reaching levels below 125 nmol/L (∼50 mg/dL) [3]. The ongoing phase 3 Lp(a) HORIZON trial (NCT04023552) has enrolled over 8300 patients with established ASCVD and Lp(a) levels of ≥70 mg/dL (approximately 149 nmol/L), and its results will clarify whether these reductions translate into reduced MACE [168]. Until then, expert prioritization for such agents remains based on clinical risk and event history. Olpasiran, zerlasiran, and lepodisiran are siRNA molecules that silence LPA gene expression within the cytosol of hepatocytes at the RNA-induced silencing complex (RISC) [169]. These agents are injected subcutaneously two to four times per year [3]. Olpasiran is currently being evaluated in the OCEAN(a) outcomes trial (NCT05581303), which has enrolled over 7000 ASCVD patients with Lp(a) ≥200 nmol/L (∼85 mg/dL) [170]. In contrast, muvalaplin represents a novel oral therapy that inhibits the extracellular covalent binding of apo(a) to apoB, thereby reducing Lp(a) assembly. Though still in earlier stages of development, it has shown Lp(a) reductions of up to 65% and may offer a non-injectable option in the future [3].

ASOs and siRNAs differ in their site of action, mechanism, and dosing frequency. ASOs typically enter the nucleus, where they bind target mRNA and induce RNase H-mediated degradation, whereas siRNAs act in the cytoplasm by incorporating into the RISC to guide mRNA cleavage [13]. These pharmacologic distinctions influence dosing schedules: ASOs generally require more frequent administration, usually monthly, while siRNAs have longer durations of action that can enable biannual dosing [171]. A figure illustrating these mechanistic and pharmacokinetic differences can be found in *Gareri* et al.*, Antisense Oligonucleotides and Small Interfering RNA for the Treatment of Dyslipidemias, Journal of Clinical Medicine (2022)* Fig. 1 *which states “mRNA degradation mechanisms through ASO and siRNA. (A) The ASO mechanism of action: The single-strand ASO enters the cell and the nucleus; once it is bound to the mRNA, the double-strand siRNA is recognized by RNAse H and degraded. (B) The siRNA mechanism of action: The double-strand siRNA enters the cell; in the cytoplasm, the duplex opens and the antisense strand binds to the RNA-induced silencing complex (RISC). The mRNA is recognized by the antisense-RISC complex and degraded”. Adapted from Gareri* et al.*, Antisense Oligonucleotides and Small Interfering RNA for the Treatment of Dyslipidemias, Journal of Clinical Medicine (2022)* [13].

Of particular clinical relevance is the ongoing effort to identify Lp(a) receptors and uptake pathways that could be targeted to reduce circulating levels. Although PCSK9 inhibitors are primarily used for LDL-C reduction, they have been shown to also lower Lp(a) concentrations. Several candidate receptors, interacting through apoB-100 or oxidized phospholipid domains, have been proposed, but a definitive Lp(a) receptor has not yet been identified. Notably, recent work has uncovered two distinct plasminogen receptor-mediated pathways facilitating Lp(a) internalization and clearance, expanding potential therapeutic avenues for targeted Lp(a) reduction [68]. A conceptual illustration of the proposed Lp(a) receptors and uptake pathways is presented in Weiss L, Aikawa E. Glycolysis hijacked: a novel pathogenic role of lipoprotein(a) in valve disease. Eur Heart J Open. 2025; 5(4):oeaf069, Fig. 1, which states “*Putative lipoprotein(a) receptors in chronological order of their discovery. Experimental studies suggest direct binding of lipoprotein(a) (-components) to these receptors. However, subsequent uptake, clearance, and/or downstream signalling mechanisms remain largely elusive.” Adapted from Weiss L, Aikawa E. Glycolysis hijacked: a novel pathogenic role of lipoprotein(a) in valve disease. Eur Heart J Open. 2025;5(4):oeaf069* [68]

Most experts (92.31%) supported a tiered strategy, reflecting a pragmatic framework for integrating current and future interventions. This includes lifestyle counseling in borderline-risk individuals, aggressive LDL-C lowering in high-risk cases, and the eventual incorporation of Lp(a)-specific therapies. The single neutral vote may reflect uncertainty about how best to implement this strategy in healthcare systems lacking robust lipid infrastructure.

LDL apheresis has demonstrated effectiveness in reducing Lp(a) by more than 30% per session [172]and is currently used as a last-resort intervention in patients with progressive ASCVD and extreme Lp(a) elevation [141,173,174]. In a retrospective study by Schumann et al., long-term lipoprotein apheresis in high-risk patients with isolated elevated Lp(a) levels led to a significant reduction in MACE. The annual MACE rate decreased from 0.34 to 0.006 events per patient per year, representing a 98% reduction. These findings support the use of lipoprotein apheresis in patients with elevated Lp(a) who continue to experience CV events despite optimal lipid-lowering therapy and lifestyle modifications [156]. While costly and resource-intensive, its temporary role in selected patients was endorsed by 84.61% of experts. The neutral and dissenting votes likely reflect logistical barriers, high cost, and the anticipation of pharmacologic alternatives that may replace this invasive modality.

All experts (100%) agreed that patients with elevated Lp(a) should receive comprehensive education about its genetic nature, associated CV risks, and the pipeline of emerging therapies. High Lp(a) levels may provoke anxiety or fatalism, and counseling on modifiable risk factors and future treatment options is critical for promoting adherence and reducing psychological distress [175].

**Fig. 4:**
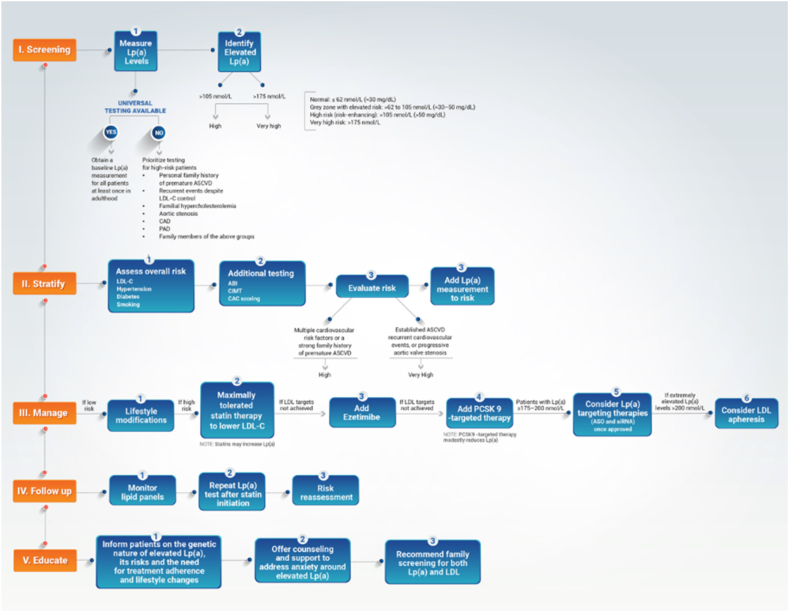
Clinical Decision-Support Flowchart for Managing Elevated Lp(a) Lp(a), lipoprotein(a); ASCVD, atherosclerotic cardiovascular disease; LDL-C, low-density lipoprotein cholesterol; CAD, Coronary Artery Disease; PAD, Peripheral Artery Disease; ABI, Ankle Brachial Index; CIMT, carotid intima-media thickness; CAC, coronary artery calcium; PCSK9, Proprotein convertase subtilisin/kexin type 9; ASO, antisense oligonucleotides; siRNA, small interfering ribonucleic acid.

With Lp(a)-targeting therapies approaching clinical use, there is a need for clear guidance on how to identify and prioritize eligible patients [176]. The following flowchart outlines a practical stepwise approach to help clinicians integrate future therapies into CV risk management (Fig. 4).

6. Primary vs. Secondary Prevention Considerations
  6.1. Primary prevention should focus on early identification of individuals with high Lp(a) levels and aggressive risk factor modification, including intensive LDL-C lowering, lifestyle interventions, smoking cessation and periodic imaging assessments such as coronary artery calcium (CAC) scoring for refined risk prediction (A = 100%, N = 0%, D = 0%)
  6.2. Secondary prevention should prompt more aggressive lipid-lowering strategies and consideration of PCSK9 targeted therapy (A = 100%, N = 0%, D = 0%)
  6.3. Referral pathways should be standardized to ensure that individuals with significantly elevated Lp(a) receive timely consultation with lipidologists, cardiologists, and endocrinologists (A = 92.31%, N = 7.69%, D = 0%)
  6.4. Imaging-guided strategies, including CAC scoring and vascular ultrasound, should be emphasized to personalize treatment decisions, particularly for patients with borderline risk (A = 84.62%, N = 15.38%, D = 0%)
  6.5. Clinical guidelines should be updated to clearly define Lp(a) thresholds for specialist referral, ensuring a streamlined approach to managing high-risk individuals and reducing variability in clinical decision-making (A = 92.31%, N = 7.69%, D = 0%)
  6.6. Insurance coverage for PCSK9 targeted therapies should be considered in patients with high Lp(a) and high cardiovascular risk (A = 76.93%, N = 23.07%, D = 0%)
  6.7. There is a need for clearer guidelines on Lp(a) management in primary prevention, particularly regarding treatment thresholds (A = 100%, N = 0%, D = 0%)
  6.8. Elevated Lp(a) is associated with persistent residual cardiovascular risk despite optimal LDL-C lowering (A = 84.62%, N = 0%, D = 15.38%)
  6.9. Statins do not lower Lp(a) and may even slightly increase its levels in some patients (A = 100%, N = 0%, D = 0%)
  6.10. PCSK9 targeted therapies reduce Lp(a) levels by approximately 15-30% but are primarily indicated for LDL-C reduction (A = 92.31%, N = 0%, D = 7.69%)
  6.11. Emerging therapies, such as antisense oligonucleotides (ASO) and small interfering RNA (siRNA), show promise in lowering Lp(a) levels by up to 90-95%, and may significantly impact cardiovascular outcomes (A = 100%, N = 0%, D = 0%)
  6.12. Until Lp(a)-lowering therapies with clinical outcome data become available, risk reduction strategies should focus on aggressive LDL-C lowering and lifestyle modifications (A = 100%, N = 0%, D = 0%)
  6.13. Niacin has modest effects on Lp(a) but is limited by poor tolerability (A = 84.62%, N = 15.38%, D = 0%)
  6.14. LDL apheresis is an option for patients with extremely high Lp(a) levels, though it is costly and requires frequent sessions (A = 84.62%, N = 15.38%, D = 0%)
  6.15. Further studies are needed to assess Lp(a) prevalence and cardiovascular risk impact in the Gulf region (A = 100%, N = 0%, D = 0%)
  6.16. Patients with metabolic syndrome, diabetes, or chronic kidney disease may have unique risk profiles requiring tailored management strategies for elevated Lp(a) (A = 84.62%, N = 15.38%, D = 0%)
  6.17. Consideration should be given to the psychological impact of a high Lp(a) diagnosis, with appropriate counseling on risk management and future therapeutic options (A = 100%, N = 0%, D = 0%)

This section of the consensus builds upon previous themes to provide a unified framework for incorporating Lp(a) into both primary and secondary prevention of ASCVD. Drawing from prior discussions on measurement, risk stratification, and therapy, the panel unanimously agreed on the need for early identification of elevated Lp(a), ideally through universal, one-time screening, as a cornerstone of primary prevention [135,137,141]. This view is strongly supported by the findings of the recent EPIC-Norfolk study, which demonstrated that elevated Lp(a), in combination with LDL-C and high-sensitivity CRP, independently and additively predicts CV events over a 20-year period, reinforcing the value of integrating these markers into preventive care [177]. In individuals with elevated Lp(a), especially those identified through screening in primary prevention settings, lifestyle interventions remain a cornerstone of care, not because they reduce Lp(a) directly, but because they significantly modify the associated CV risk. The EPIC-Norfolk prospective population study, which followed 14,051 adults over 11.5 years, found that although elevated Lp(a) levels were associated with increased CV risk, this risk was substantially attenuated in individuals who achieved ideal CV health, defined using the American Heart Association's metrics—non-smoking status, healthy body mass index, regular physical activity, a healthy diet, normal blood pressure, optimal fasting glucose, and controlled total cholesterol. Among participants with genetically high Lp(a) levels, those who had ideal health metrics had lower risk of CVD compared to those with poor health metrics [70]. This underscores that even in the presence of a non-modifiable risk factor like Lp(a), aggressive risk factor optimization remains a powerful strategy for both primary and secondary prevention.

While LDL-C lowering remains essential, particularly in secondary prevention, several studies have shown that the risk associated with elevated Lp(a) persists despite optimal lipid control, including with statin or PCSK9 targeted therapy, underscoring the concept of residual risk [74,113,160,178]. Importantly, this does not diminish the central role of aggressive LDL-C and apoB lowering as the cornerstone of cardiovascular risk reduction, but rather highlights the presence of persistent Lp(a)-mediated residual risk that may require additional targeted approaches. Multiple trials have demonstrated that statins may modestly increase Lp(a) levels in a subset of patients, an unintended effect that has been consistently reported in both observational cohorts and randomized controlled trials [79,124,179]. These findings further underscore the need for therapies that specifically target Lp(a), such as ASOs and siRNAs, which have shown significant reductions in early-phase trials and are currently being evaluated in large CV outcome studies [3,161]. Recognizing the persistence of residual risk, the panel recommended incorporating imaging modalities such as CAC scoring and vascular ultrasound, particularly for individuals with borderline or discordant risk profiles. Additional consensus emerged around the importance of establishing clear referral pathways, updating guidelines with specific Lp(a) thresholds, and ensuring equitable access to future therapies [138]. However, disparities in voting around insurance coverage and interventions like LDL apheresis reflect practical uncertainties, especially in health systems with limited access to lipid clinics or specialist care [156]. Notably, this section also reaffirms the need to address the psychological burden of an elevated Lp(a) diagnosis and to provide structured patient education around modifiable risk factors and emerging therapies [175]. Finally, a major gap remains in the applicability of global evidence to the Middle East and particularly the Gulf region. There is an urgent need for population-based studies that characterize Lp(a) prevalence, ethnic variability, and CV risk in Arab populations. Existing risk calculators developed in Western cohorts may underestimate risk in this setting, and guideline development must incorporate region-specific data to ensure appropriate stratification and intervention. Without such data, regional clinicians are left to extrapolate from international studies, which may not reflect the genetic, demographic, or healthcare realities of their patients. This consensus thus highlights a dual need: implementing best available global evidence while actively working to generate region-specific insights to guide future practice.

Bringing Lp(a) testing into routine clinical care across the Gulf region starts with identifying the right patients and establishing practical referral pathways. In primary care, Lp(a) should be considered for individuals with a strong family history of premature CV disease, recurrent events despite optimal lipid control, or known familial hypercholesterolemia, a profile that reflects many patients in the region. These indications are in line with international guidance recommending a one-time lifetime measurement in high-risk individuals [23]. The 2025 ESC/EAS focused update highlights that CV risk associated with Lp(a) begins to rise modestly at concentrations around 62 nmol/L (≈30 mg/dL) and becomes clinically meaningful above 105 nmol/L (≈50 mg/dL), with progressively higher levels conferring greater risk [80]. Because elevated Lp(a) contributes incrementally to ASCVD risk, failure to account for it may result in underestimation of overall CV risk. Accordingly, when Lp(a) concentrations exceed 62 nmol/L (≈30 mg/dL), patients should be referred to specialized lipid or cardiology clinics for comprehensive risk evaluation and management. In healthcare systems where access to lipid specialists is constrained, endocrinologists or internal medicine physicians may serve as key partners in referral and follow-up care.

For many patients, receiving an elevated Lp(a) result can be unsettling, particularly given the genetic nature of the condition and the perception that little can be done. It is essential to explain that Lp(a) is inherited and not caused by diet or lifestyle, and that having high levels does not mean CVD is inevitable. Instead, the focus should be on managing modifiable risk factors: ensuring LDL-C is aggressively lowered, controlling blood pressure and diabetes, and using imaging tools like coronary artery calcium scoring or carotid ultrasound to better understand actual risk. It is also helpful to reassure patients that promising new therapies aimed specifically at lowering Lp(a) are in advanced stages of development [6]. Providing this context helps patients stay engaged and less anxious, and positions primary care as a critical entry point in the region's broader CV prevention efforts [180].

To support clinical decision-making, we propose a structured framework outlining when to test for Lp(a), thresholds for specialist referral, and treatment approaches tailored to primary and secondary prevention settings. Table 2 synthesizes expert consensus and current evidence, and is intended to guide clinicians in the Gulf region in applying consistent, risk-based strategies, particularly as emerging Lp(a)-lowering therapies become available.

**Table 2:** Structured clinical guidance for Lp(a) management in primary and secondary prevention.

Category	Primary Prevention	Secondary Prevention
Patient Population	Individuals with family history of premature ASCVD, FH, or borderline traditional risk scores	Patients with established ASCVD (Ischemic Myocardial Infarction, Stroke, or Peripheral Arterial Disease) recurrent CV events, or aortic valve stenosis
When and Whom to Test for Lp(a)	At least once in adulthood for high-risk individuals or those with strong family history	During baseline risk reassessment or in persistent residual risk despite therapy
Referral Threshold	Lp(a) ≥ 62 nmol/L with other risk factors (INCREASED RISK) Lp(a) ≥ 105 nmol/L despite optimal LDL-C lowering (HIGH RISK)	Lp(a) ≥ 62 nmol/L with other risk factors (INCREASED RISK) Lp(a) ≥ 105 nmol/L despite optimal LDL-C lowering (HIGH RISK)
Initial Management	Lifestyle modification, LDL-C and apoB reduction, CAC scoring to refine risk	Ensure maximally tolerated statins and ezetimibe; intensify risk control
Advanced Therapies	Consider PCSK9 targeted therapy (combined with lipid-lowering therapy) if LDL-C remains elevated; ASO/siRNA when available for high Lp(a)	Add PCSK9 targeted therapy; prioritize for ASO/siRNA when approved; consider LDL apheresis in patients with recurrent cardiovascular events
Follow-Up Plan	Annual lipid review; re-image or retest if risk profile changes or new therapies become available	Reassess lipids every 6-12 months; monitor for event recurrence; update plan as new options emerge

## 4 Limitations

Several limitations should be acknowledged. First, although the expert panel included experienced clinicians from multiple specialties and several Gulf countries, representation was limited to four countries (United Arab Emirates, Kuwait, Bahrain, and Oman). Experts from other Gulf states were not included, which may limit the generalizability of some recommendations across the entire region. Nevertheless, the participating panelists have extensive clinical experience managing dyslipidemia and cardiovascular risk in Gulf populations, and the consensus statements were formulated with consideration of shared regional healthcare characteristics and epidemiological patterns.

Second, this consensus was developed using a modified Delphi methodology that included a pre-meeting questionnaire, structured expert discussion, and a single round of anonymous voting. While classical Delphi processes typically involve multiple iterative rounds, the preparatory questionnaire and expert discussions allowed refinement of candidate statements prior to voting. Consequently, a single voting round was used to confirm agreement among panelists. However, the absence of multiple rounds may limit opportunities for further refinement and could affect the robustness and reliability of consensus compared with traditional multi-round Delphi approaches.

Finally, although the statements were informed by an extensive review of the available literature, some recommendations necessarily reflect expert clinical judgment due to the limited availability of region-specific data on Lp(a) prevalence, risk stratification, and therapeutic strategies in Gulf populations. Additional epidemiological and clinical studies in Middle Eastern populations will be essential to validate and refine these recommendations.

## 5 Conclusion

As a primarily genetically determined, lifelong CV risk factor, Lp(a) presents unique challenges and opportunities in the prevention and management of ASCVD. This Gulf-based expert consensus synthesizes current evidence and clinical experience to offer regionally relevant guidance, emphasizing the urgent need for greater awareness, standardized testing protocols, and equitable access to emerging therapies. While many insights align with global recommendations, the distinct genetic, demographic, and healthcare characteristics of the Middle East and Gulf region demand tailored approaches. As research evolves and Lp(a)-targeted treatments approach clinical availability, this consensus provides a timely foundation to inform future guideline development and improve CV outcomes across the region.

## Author contributions

Wael Almahmeed and Hani Sabbour contributed equally to this work. They led the conceptualization and coordination of the expert panel, oversaw the modified Delphi process, contributed substantially to the literature review, data interpretation, and drafting of the manuscript, and share equal responsibility for the overall content.

Ronney Shantouf, Asma Aljaberi, Farhana Bin Lootah, Nasreen Al Sayed, Juwairia AlAli, Mousa Akbar, Abdullah Shehab, Hassan El Tamimi, Ahmad AlSarraf, and Khalid Al Waili served as members of the expert panel, providing clinical insights based on regional experience, participating in voting, and contributing to the interpretation of consensus findings.

Thomas Lüscher provided critical oversight of the survey design and methodology, and offered expert guidance throughout the drafting and revision process.

All authors critically reviewed, edited, and approved the final version of the manuscript.

The other authors declare that they have no known competing financial interests or personal relationships that could have appeared to influence the work reported in this paper.

## Data statement

This article does not include any primary patient-level data. The work is based on expert consensus and anonymized aggregated voting data. The underlying materials (e.g., questionnaire items and summary voting results) are available from the corresponding author upon reasonable request.

## Declaration of generative AI and AI-assisted technologies in the manuscript preparation process

During the preparation of this work, the authors used AI-assisted tools to support grammar, spelling, and reference checking. The authors subsequently reviewed and edited the manuscript and take full responsibility for the accuracy and integrity of the content.

## Funding

This work was supported by 10.13039/100004336Novartis (for medical writing support and meeting logistics). However, Novartis has not influenced the publication's content or been involved in its writing.

## Declaration of competing interest

The authors declare the following financial interests/personal relationships which may be considered as potential competing interests: Hani Sabbour reports financial support was provided by Novartis Pharmaceuticals. Thomas F. Lüscher has outside this work received research and educational grants to the institution from the Swiss Heart Foundation, the Swiss National Research Foundation, the Foundation for Cardiovascular Research, the European Union (Horizon) and from industry, i.e. Abbott, Amgen, Alynam, AstraZeneca, Bayer Healthcare, Boehringer-Ingelheim, Cytokinetics, Daichi-Sankyo, Eli Lilly, Novartis, Novo Nordisk, Pfizer, Roche Diagnostics, Sanofi and Vifor. He no longer accepts honoraria from industry partners. The other authors declare that they have no known competing financial interests or personal relationships that could have appeared to influence the work reported in this paper.

If there are other authors, they declare that they have no known competing financial interests or personal relationships that could have appeared to influence the work reported in this paper.
